# Carbapenem-Resistant *Acinetobacter baumannii:* Virulence Factors, Molecular Epidemiology, and Latest Updates in Treatment Options

**DOI:** 10.3390/microorganisms13091983

**Published:** 2025-08-26

**Authors:** Theodoros Karampatakis, Katerina Tsergouli, Payam Behzadi

**Affiliations:** 1 Department of Clinical Microbiology, University Hospital Kerry, V92 NX94 Tralee, Ireland; aikaterini.tsergouli@hse.ie; 2 Department of Microbiology, ShQ.C., Islamic Azad University, Shahr-e Qods 37541-374, Iran

**Keywords:** carbapenem-resistant *Acinetobacter baumannii*, molecular epidemiology, antimicrobial agents, virulence factors, antimicrobial treatment

## Abstract

*Acinetobacter baumannii* is a Gram-negative, non-motile pathogen commonly associated with healthcare settings. It is capable of causing severe infections, particularly in immunocompromised and critically ill individuals, and is linked to poor clinical outcomes. Infections caused by carbapenem-resistant *A. baumannii* (CRAB) represent a major public health concern due to limited treatment options and high resistance rates. Several virulence determinants contribute to CRAB’s pathogenicity, including capsular exopolysaccharide (CPS), lipopolysaccharide (LPS), lipooligosaccharide (LOS), efflux pumps, outer membrane proteins (OMPs), pili, metal acquisition systems, two-component regulatory systems (TCSs), and secretion systems (SSs). The dominant resistance mechanism in CRAB involves the production of carbapenemases, most notably oxacillinase-23 (OXA-23) and metallo-β-lactamases (MBLs) such as Verona integron-encoded MBL (VIM) and New Delhi MBL (NDM). Accurate identification of these resistance mechanisms is crucial for guiding effective antimicrobial therapy. Potential treatment options include older agents like polymyxins, ampicillin–sulbactam, high-dose carbapenems, tigecycline, and minocycline, along with newer antimicrobials such as eravacycline, cefiderocol, and aztreonam–avibactam. This review aims to explore the virulence mechanisms and molecular pathogenesis of CRAB, while also presenting recent developments in its epidemiology and available antimicrobial therapies.

## 1. Introduction

*Acinetobacter baumannii* is a Gram-negative, non-motile pathogen frequently encountered in healthcare environments, where it is responsible for severe infections, particularly in critically ill or immunocompromised patients [1]. Its presence in intensive care units (ICUs) is of particular concern, as carbapenem-resistant *A. baumannii* (CRAB) has been linked to higher rates of mortality and morbidity, as well as substantial healthcare costs [1,2,3]. CRAB emergence is primarily driven by cross-transmission, often resulting from lapses in infection control measures—such as poor hand hygiene—and extensive use of antimicrobials, which fosters selective pressure for resistant strains [4,5]. The 2024 World Health Organization (WHO) Bacterial Priority Pathogens List (BPPL) designates CRAB, third-generation cephalosporin-resistant Enterobacterales, carbapenem-resistant Enterobacterales (CRE), and rifampicin-resistant *Mycobacterium tuberculosis* as pathogens of critical priority [6]. The burden of CRAB is especially severe in endemic areas [7], with Greece reporting the highest prevalence among EU countries, reaching a resistance rate of 95.3% in 2023 [8]. CRAB isolates are commonly categorized into three resistance levels: multidrug-resistant (MDR), defined as non-susceptibility to at least one agent in three antimicrobial classes; extensively drug-resistant (XDR), defined as non-susceptibility to at least one agent in all but two classes; and pandrug-resistant (PDR), characterized by non-susceptibility to all agents across all tested categories [9,10,11,12,13]. Recent studies show that most CRAB isolates remain susceptible to only one or two available antibiotics, placing them in the XDR category. In some cases, these strains further progress to PDR status, leaving no viable treatment options. The persistence and spread of XDR/PDR *A. baumannii* within healthcare environments are linked to elevated mortality rates and substantial infection control challenges, highlighting the urgent need for innovative and effective antimicrobial approaches [14,15,16,17]. Thus, a comprehensive understanding of both the resistance mechanisms at the molecular level and the epidemiological trends of CRAB is critical for developing and selecting effective treatment strategies [18]. Accordingly, this review aims to highlight the virulence traits of CRAB and provide updated insights into its molecular epidemiology and current therapeutic options.

## 2. Genomic Pool, Virulence Factors, and Molecular Pathogenesis

The genome of *A. baumannii* consists of a core (persistent) component, present in 95–99% of strains, which encodes essential functions such as cell division, metabolism, and energy production [19,20,21]. In contrast, the accessory (shell) genome, found in 15–35% of isolates, contains genes related to virulence and antimicrobial resistance, often acquired via mobile genetic elements (MGEs) like plasmids, transposons (Tns), and integrons (Ints) [20,21,22,23,24]. CRAB exhibits an open pan-genome, enriched with insertion sequences (ISs), Ints, and Tns embedded in genomic islands (GIs), facilitating the acquisition of new genes [25,26]. Antimicrobial Resistance Genes (ARGs) are distributed across both the core and shell genome, frequently mobilized by ISs and other elements through integrase and transposase activity [11,12,21,24,27,28].

Notably, *ISAba*-type ISs, approximately 1 kb in size, play a central role in activating and disseminating carbapenemase genes, significantly contributing to the genomic plasticity and multidrug resistance seen in CRAB strains [21,24,26,29,30].

The development of antimicrobial resistance in *A. baumannii* and CRAB strains is largely driven by horizontal gene transfer (HGT), alongside notable contributions from mutations and the MGEs, particularly those of the *ISAba* family. Such genomic changes can influence the expression of constitutive genes involved in enzyme synthesis, efflux pump function, and outer membrane protein (OMP) modification, thereby equipping the bacteria with resistance to a wide spectrum of antimicrobial agents [21,24,31].

The GI AbaR1 plays a pivotal role in acquiring a wide array of resistance determinants through its multiple class 1 Ints in *A. baumannii* and CRAB strains. These Ints carry gene arrays capable of expressing a diversity of effective enzymes, e.g., β-lactamases, aminoglycoside-modifying enzymes, and efflux systems. Expression of these enzymes results in the appearance of resistance in CRAB superbugs to not only numerous antimicrobial agents (such as sulfonamides, chloramphenicol, trimethoprim, etc.) but also a variety of heavy metals and some other chemicals. In addition to AbaR1, the proliferation of ARGs in *A. baumannii* and CRAB strains is further facilitated by plasmid-mediated HGT [24,31,32].

Plasmids serve as major mediators of HGT, playing a central role in the dissemination of carbapenem-resistant genes (CRGs), particularly within CRAB populations. It is known that the *bla*_OXA-58_ gene is frequently plasmid-borne, and studies have reported instances where *bla*_NDM-1_ and *bla*_OXA-58_ coexist on the same large plasmid [31,33,34]. Although uncommon in *A. baumannii*, metallo-β-lactamase (MBL) genes—such as *bla*_NDM_, *bla*_VIM_, and *bla*_IMP_—as well as Class A β-lactamase genes—such as *bla*_KPC_ and *bla*_GES_—have been identified on plasmids. These plasmid-borne genes have been reported in *A. baumannii*, e.g., CRAB strains [35]. In a study, Ramoul et al. documented the co-existence of *bla*_NDM-1_ alongside *bla*_OXA-23_ or *bla*_OXA-58_ in clinical *A. baumannii* isolates obtained from northeastern Algeria [31,36].

In a comparative analysis, Wang et al. [37] investigated the MGEs of two strains comprising 2023-AB023 and 2023-AB033. Strain 2023-AB023 harbored six plasmids, while strain 2023-AB033 carried a subset of four of these. At the chromosomal level, both strains shared five common MGEs, e.g., *ISAba8*, *ISAlw1*, *ISAba21*, *ISAba26*, and *ISAba14*.

They [37] also found that the ARGs of *bla*_OXA-23_ and *bla*_NDM-1_ were located on separate plasmids in each strain, while all other ARGs were situated on the chromosome. In addition, the *bla*_NDM-1_-carrying plasmid was linked to the *ISAba125*, which encodes the *IS3* family transposase *ISAba45*.

CRAB expresses a wide array of virulence factors, categorized into nine major types: capsular polysaccharide (CPS), lipopolysaccharide (LPS), lipooligosaccharide (LOS), efflux systems, OMPs, pili, metal ion acquisition systems, two-component regulatory systems (TCSs), and secretion systems (SSs) [12,21,38]. Most of the genes responsible for these virulence traits are disseminated via MGEs—such as ISs, Tns, and Ints—frequently carried on plasmids [39,40].

### 2.1. Capsular Exopolysaccharide (CPS)

The high-molecular-weight polysaccharide capsule is essential for immune evasion and environmental persistence. It supports growth in ascitic fluid, resists complement killing, and is critical for virulence in pulmonary infection models. *A. baumannii* exhibits phase variation between highly capsulated (virulent) and low-capsulated (avirulent) forms. This switch is regulated by TetR-family transcription factors, affecting susceptibility to antimicrobials, lysozyme, disinfectants, and oxidative stress. Genes like *wzc* and small RNAs such as SrvS influence this phenotypic shift [41,42,43,44].

The capsular type (K-type) is a key determinant of *A. baumannii* and CRAB virulence, as the capsule provides vital defense against environmental challenges, nutrient limitation, and host immune responses. Driven by its crucial protective role and intense selective pressures, the capsule exhibits remarkable structural diversity, with over 200 distinct K loci identified to date [45,46,47,48]. Unlike KL2, the most prevalent K-type worldwide, many other K-types display regional distribution patterns. For instance, KL2 and KL7 have been reported in CRAB strains from Portugal, KL7 and KL28 in China, and KL9 in Canada. In Taiwan, a wider diversity has been observed, including KL2, KL10, KL14, KL22, and KL52 [46,48,49,50,51].

### 2.2. Lipopolysaccharide (LPS)

LPS is a key outer membrane component of Gram-negative bacteria, consisting of lipid A, a core oligosaccharide, and the O-antigen. In *A. baumannii*, LPS contributes to immune evasion and triggers host inflammatory responses [52,53,54,55,56,57]. Lipid A, the toxic portion, anchors the LPS and modulates membrane permeability and antibiotic resistance via enzymatic modifications during biosynthesis. Its hepta-acylated form is predominant in *A. baumannii*, supporting membrane integrity through divalent cation bridges and negative charge interactions. Specific sugar compositions and structural variations, such as mutations in lipid A biosynthesis genes (e.g., *lpxABCD*), have been linked to resistance against polymyxins [38,55,58].

### 2.3. Lipooligosaccharide (LOS)

*A. baumannii* expresses various surface structures—including adhesins, capsular polysaccharides, glycoproteins, and LOS—that contribute to its virulence. Unlike many Gram-negative bacteria, *A. baumannii* lacks O-antigen and instead produces LOS with a hepta-acylated lipid A linked to an oligosaccharide core. This structure triggers innate immune responses via Toll-like receptor 4 (TLR4) and enhances resistance to antimicrobial peptides and desiccation. Under cold stress, the bacterium modifies lipid A using the LpxS enzyme to maintain membrane fluidity [41,59,60,61].

Wang et al. [37] found that all CRAB strains they examined possessed a core set of nine virulence genes, which are crucial for adherence (*ompA*), drug efflux (*AdeFGH*), and iron sequestration (*barA*). A notable exception was a group of six strains involving 2019-AB015, 2019-AB016, 2019-AB019, 2019-AB055, 2023-AB023, and 2019-AB023, which were missing the biofilm gene *bap* and the quorum-sensing genes *abaI/R*. [37]. The isolates showed significant genetic diversity, with 12 different KL types and three lipooligosaccharide outer core (OCL) types. The most common combinations were KL2-OCL1, KL9-OCL1, and KL3-OCL1. Interestingly, the KL30 locus was present only in the isolates collected in 2023.

### 2.4. Efflux Pumps

*A. baumannii* employs broad-spectrum resistance–nodulation–division (RND) efflux pumps to expel diverse antimicrobials. The main characterized systems—AdeABC, AdeFGH, and AdeIJK—consist of an inner membrane transporter, a membrane fusion protein, and an outer membrane channel. The AdeABC pump, regulated by the adeRS two-component system, is strongly associated with carbapenem resistance, especially when adeA, adeB, and adeC are overexpressed. Notably, carbapenem resistance can occur even in the absence of regulatory mutations, suggesting multifactorial gene interactions. Synergism between AdeABC overexpression and carbapenemase (e.g., OXA enzymes) further amplifies resistance [62,63,64,65,66,67,68].

### 2.5. Outer Membrane Proteins (OMPs)

OMPs are crucial for *A. baumannii* pathogenicity and antibiotic resistance. Among them, OmpA is the most dominant, aiding in drug resistance, adherence to epithelial cells, and biofilm formation. It interacts with host fibronectin to facilitate cell attachment and invasion, while also contributing to immune evasion, serum resistance, apoptosis induction, and outer membrane vesicle (OMV) production. OmpA’s role in modulating host immune responses, particularly by promoting Th1 differentiation via dendritic cell activation, further enhances the bacterium’s persistence and virulence [69,70,71,72,73,74,75,76].

Indeed, OMVs play multifaceted roles in bacterial physiology, with a significant contribution to cellular detoxification. By facilitating the removal of toxic periplasmic substances, OMVs alleviate envelope stress and support bacterial fitness. This detoxification capability has been observed, for example, in non-native bacterial hosts expressing Class B β-lactamases such as VIM-2 and SPM-1 [77,78].

### 2.6. Pili

*A. baumannii*’s persistence in healthcare settings is largely due to its strong adhesion capacity. Adhesion to host or abiotic surfaces is a crucial first step in infection. This process is facilitated by surface structures like pili and fimbriae, which enable firm attachment and biofilm formation. Fimbrial adhesins promote motility and surface colonization, while non-fimbrial adhesins bind host extracellular matrix components like fibronectin and collagen. These structures also aid in plasmid transfer, contributing to the spread of antibiotic resistance [79,80,81,82,83,84,85,86,87].

Adhesins are key virulence factors that help bacteria resist mechanical stress from bodily fluids. In *A. baumannii*, they include pili proteins, autotransporters, and other surface enzymes, each with distinct receptor affinities. Their interactions with host molecules—such as integrins, selectins, and TLRs—trigger immune responses and enable bacterial survival, inflammation, and biofilm stability. Targeting these adhesins could support new infection-control strategies [79,88,89,90,91,92,93]. All in all, *A. baumannii* possesses various types of pili, including chaperone-usher (CUP), curli, type IV, type V, and conjugative type IV secretion pili. Among them, CUP pili are the most common and play a key role in surface attachment during infection [10,87,94,95,96].

### 2.7. Metal Ion Uptake Systems

*A. baumannii* uses siderophores like acinetobactin to acquire iron under host-imposed limitations. Transporters BauA and BauE import iron–siderophore complexes, with Oxa binding iron directly and Isox aiding under stress. Genes like *bauB* and *bauC* drive siderophore synthesis, while the Fur regulator suppresses this process in iron-rich conditions to maintain homeostasis [97,98,99,100].

Calprotectin, released by neutrophils, restricts zinc and manganese during infection, indirectly exacerbating *A. baumannii*’s iron limitation. Alongside host iron-binding proteins like transferrin and lactoferrin, this creates a nutrient-starved environment. In response, *A. baumannii* enhances siderophore production and activates metal transporters and enzymes like ZrlA to sustain survival under metal stress [97,101,102,103]. Moreover, zinc is vital for *A. baumannii*, particularly for activating enzymes like MBLs. Overexpression of *znuABC* enhances zinc uptake from the environment, supporting bacterial survival and resistance [104].

### 2.8. Two-Component Systems (TCSs)

TCSs consist of a membrane-bound sensor and a cytoplasmic response regulator. They enable bacteria to adapt to changing environmental conditions. *Acinetobacter baumannii* possesses up to 20 distinct TCSs, which play key roles in its resistance to antibiotics, virulence, and pathogenicity by modulating gene expression in response to environmental stimuli [38,105]. TCSs like BfmRS, PmrAB, and GacSA, along with stringent response regulators such as ppGpp and DksA, play crucial roles in controlling virulence-related genes in *Acinetobacter baumannii*. The expression of these genes and associated virulence factors is highly complex. Notably, PmrAB and BfmRS influence the production of outer membrane vesicles (OMVs), and the levels of AbOmpA within these OMVs can fluctuate based on culture conditions or regulatory input from TCSs [74,106,107,108,109,110].

### 2.9. Secretion Systems (SSs)

Although six secretion systems are well-known in Gram-negative bacteria, their functions in *A. baumannii* are still under investigation. These systems contribute to virulence and antibiotic resistance. With growing resistance, carbapenems are becoming less effective against MDR strains. As a result, CRAB is a WHO-designated critical threat. Targeting secretion systems may offer new therapeutic and vaccine strategies against drug-resistant *A. baumannii* [21,111,112].

## 3. Antimicrobial Resistance Mechanisms in CRAB

**Figure 1:**
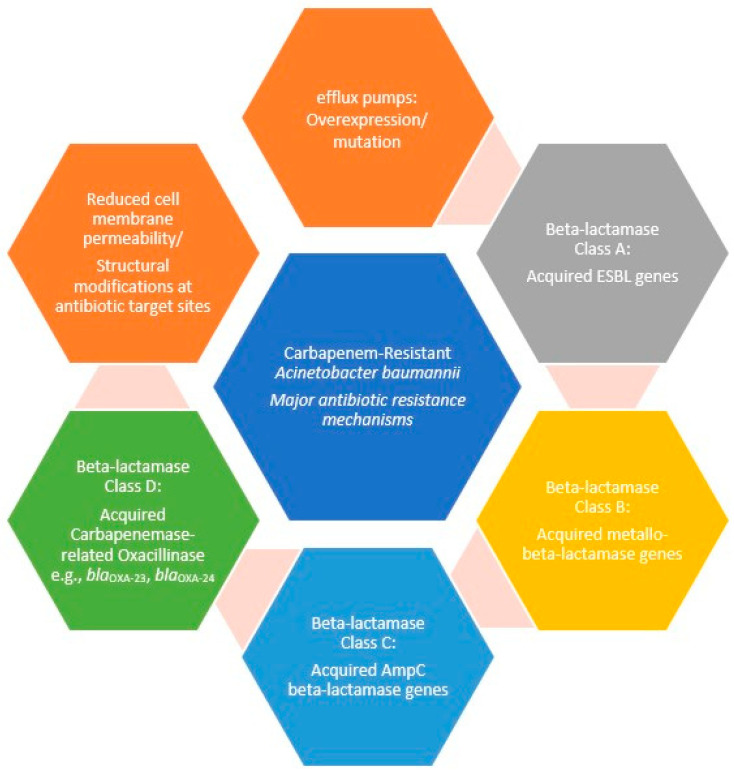
The major antibiotic resistance mechanisms in CRAB strains.

Multiple strategies are employed to resist antimicrobial agents (Figure 1), including:

(1) The production of enzymes like β-lactamases and aminoglycoside-modifying enzymes, which are normally acquired [113,114,115].
(2) Reduced cell membrane permeability due to the loss of OMPs or porins, which are normally acquired [115,116,117].
(3) Overexpression/mutation of efflux pumps, belonging to six known multidrug resistance (MDR) families, MATE, ABC, PACE, MFS, SMR, and RND, which are normally acquired [115,118].
(4) Structural modifications at antibiotic target sites, which are normally acquired [115,119].

### 3.1. Ambler Classification of β-Lactamases

Although β-lactamases were initially classified by Bush et al. [120] based on their substrate and inhibitor profiles, the molecular classification proposed by Ambler is now more widely accepted. Ambler’s system relies on amino acid sequence analysis to establish a molecular phylogeny and divides β-lactamases into four classes: A, B, C, and D [121,122].

Class A includes serine-based β-lactamases such as SHV (sulfhydryl variable), TEM (temoniera), CTX-M (cefotaxime-hydrolyzing), PER (Pseudomonas extended-resistant), GES (Guiana extended-spectrum), VEB (Vietnamese extended-spectrum), IBC (integron-borne cephalosporinase), SFO (from *Serratia fonticola*), BES (Brazil extended-spectrum), BEL (Belgium extended-spectrum), and TLA (Tlahuicas Indians). Some, like SHV, TEM, and CTX-M, can function as extended-spectrum β-lactamases (ESBLs) following minor genetic changes. This class also includes inhibitor-resistant variants such as IRTs (inhibitor-resistant TEMs), IRSs (inhibitor-resistant SHVs), and KPCs (*Klebsiella pneumoniae* carbapenemases) [122,123,124,125]. Notably, KPC-producing strains serve as long-term reservoirs, facilitating the spread of MGEs carrying the *bla*_KPC_ gene [126].

Class B β-lactamases, known as MBLs, are carbapenemases that can hydrolyze nearly all β-lactams except aztreonam. Their enzymatic activity depends on zinc ions (Zn^2+^). The most common MBLs include Verona integron-encoded MBL (VIM) and New Delhi MBL (NDM), followed by others such as imipenemase (IMP), German imipenemase (GIM), São Paulo MBL (SPM), Seoul imipenemase (SIM), Australian imipenemase (AIM), Dutch imipenemase (DIM), and the more recently identified Tripoli MBL (TMB) and Florence imipenemase (FIM) [122,127,128,129,130,131,132,133,134,135]. MBLs are further categorized into three subgroups, B1, B2, and B3 [121,122].

Class C β-lactamases, also referred to as cephalosporinases or AmpC β-lactamases, are serine-based enzymes capable of hydrolyzing a wide range of β-lactam antibiotics. Their expression can be either constitutive or inducible, and they may be encoded chromosomally or on plasmids—plasmid-encoded variants are commonly known as AmpC-like β-lactamases. Based on Jacoby’s classification, these enzymes are divided into specific subclasses according to their molecular structure and functional properties [122,136]. At present, ADC-68 is the sole Class C β-lactamase identified in *A. baumannii* that has been experimentally verified to possess carbapenemase activity [115,122,137].

Class D β-lactamases, known as oxacillinases (OXA), are also serine-based enzymes. Their hydrolytic activity varies by subtype, with certain subtypes functioning as carbapenemases and contributing to broad-spectrum resistance and multidrug-resistant phenotypes. Most OXA-type carbapenemases have been identified in CRAB, including the recently emerging OXA-48 variant [122,138,139,140]. The majority of OXA-type carbapenemase genes are known as acquired carbapenemase genes (ACGs) in CRAB strains [115,122].

*A. baumannii* naturally harbors the *bla*_OXA-51_ gene, which typically exhibits low carbapenemase activity. However, its expression can be markedly enhanced by the presence of upstream IS elements [115].

According to an in silico analysis conducted by Capodimonte et al. [78] in a Dry Lab [141], enzymes belonging to Classes A, B, and C exhibited a very low incidence of lipobox sequences. Conversely, Class D β-lactamases, primarily composed of OXA types, showed a significantly higher prevalence, with roughly 60% possessing a lipobox sequence, leading to the inference of their likely function as membrane-associated lipoproteins. Indeed, these predicted OXA β-lactamase lipoproteins were exclusively detected within *Acinetobacter* spp. [78]. Moreover, Capodimonte et al. [78] demonstrated that OXA β-lactamases are membrane-associated, a characteristic that facilitates their incorporation into OMVs, akin to NDM-1. This tendency is further supported by predictions indicating that the major carbapenem-hydrolyzing Class D β-lactamases (CHDLs)—including the chromosomally encoded OXA-51-like and the acquired OXA-23-like, OXA-58-like, and OXA-24/40-like variants—function as lipoproteins [78]. Furthermore, according to Capodimonte et al. [78], soluble OXA enzymes are not expected to exist in *Acinetobacter* spp. While lipidated Class A and B enzymes are prevalent across many bacterial hosts, the direct association of protein lipidation with a specific host like *Acinetobacter* is a characteristic unique to Class D enzymes [78].

Migliaccio et al. [142] conducted a phylogenomic study involving 837 *Acinetobacter* isolates representing 72 different species. Using the Pasteur Multilocus Sequence Typing (MLST) scheme, they analyzed phylogenetic relationships and compared them with genome-based and ribosomal MLST (rMLST) phylogenies within the *A. baumannii* group. Additionally, the study aimed to identify ARGs across the *Acinetobacter* genomes [142]. ARGs associated with at least three different antibiotic classes were detected in 91 isolates spanning 17 distinct *Acinetobacter* spp. Moreover, a class D oxacillinase—an enzyme inherently found in various *Acinetobacter* spp.—was detected in 503 isolates spanning 35 different species within the genus [142]. Moreover, Migliaccio et al. identified class D oxacillinases in 503 isolates across 35 *Acinetobacter* spp. A total of 94 class D β-lactamase genes from 11 *bla*_OXA_ families—including *bla*_OXA-211, -134, -214, -294, -51, -213, -274, -286, -58, -40_, and _-23_—were detected. Within the *A. baumannii* group, *A. baumannii* and *A. pittii* carried intrinsic *bla*_OXA-51_ and *bla*_OXA-213_, respectively, while *A. nosocomialis* and *A. seifertii* lacked intrinsic class D β-lactamases.

Carbapenem resistance in *A. baumannii* has increased globally, largely due to acquired class D β-lactamases—*bla*_OXA-23_, *bla*_OXA-40_, and *bla*_OXA-58_. Among them, *bla*_OXA-23_, originally identified in Scotland and likely derived from *A. radioresistens*, is the most widespread. Outbreaks of OXA-23-producing strains have been reported in various countries, from Eastern Europe and West Asia to Southeast Asia and South America [143,144,145,146].

The spread of *bla*_OXA-23_ is linked to Tns, e.g., *Tn2006*, *Tn2007*, and *Tn2008*. *Tn2006* includes two *ISAba1* elements, while *Tn2008* has one. *Tn2007* carries a single ISAba4. In some strains reported from the Arab states of the Persian Gulf region, the gene is related to one *ISAba1* [143,147,148,149,150,151,152].

According to Papadopoulou et al.’s report [17], all examined *A. baumannii* isolates possessed an intrinsic, chromosomally located *bla*_OXA-51-like_ gene. In 97.7% of the isolates, a *bla*_OXA-23-like_ gene was present. Those isolates harboring *bla*_OXA-23-like_ demonstrated resistance to carbapenems. In these strains, an *ISAba1* element was situated upstream of the gene’s promoter region, a genetic arrangement that may enhance OXA-23 expression [17,153,154].

Accordingly, Mugnier et al. [143] investigated factors contributing to the global spread of the *bla*_OXA-23_ gene in *A. baumannii*. They analyzed 20 OXA-23-producing CRAB strains from various regions (including Asia and Europe) using pulsed-field gel electrophoresis and MLST. Eight distinct sequence types were identified, including four novel ones. The majority of isolates were associated with two dominant European clonal lineages [143].

### 3.2. Reduced Cell Membrane Permeability Due to the Loss of OMPs

OMP loss contributes secondarily to the development of CRAB. Studies have linked the loss of the CarO porin to imipenem resistance [155,156]. Additionally, overexpression of iron-regulated OMPs under iron-limiting conditions may further enhance carbapenem resistance [157]. The connection between the absence of the 29-kilodalton Omp and reduced susceptibility to imipenem has also been recognized for years [158].

### 3.3. Overexpression of Efflux Pumps

Efflux pump overexpression also plays a secondary role in the emergence of CRAB. Mutant-based experimental models have highlighted the significant contribution of the AdeABC efflux pump to carbapenem resistance [159]. Additionally, efflux pumps belonging to the RND family have been linked to imipenem resistance in *A. baumannii* [160].

### 3.4. Structural Modifications at Antibiotic Target Sites

Scientific evidence on target site modifications contributing to antimicrobial resistance in *A. baumannii* remains limited. The most commonly reported mechanism involves reduced binding affinity of penicillin-binding protein 2 (PBP2) for imipenem [161]. These alterations are typically caused by genetic mutations at specific loci or by post-transcriptional modifications of certain proteins [162].

## 4. Trends in Molecular Epidemiology

The earliest reports of CRAB emerged as isolated cases several years ago [163,164]. Initially, the carbapenem resistance was linked to MBLs and OXA-type enzymes [164]. However, in the early stages of CRAB’s genetic evolution, some strains exhibited low-level resistance to carbapenems despite the absence of detectable carbapenemase production [165].

### 4.1. OXA-Type Carbapenemases

The chromosomally encoded *bla*_OXA-51-like_ gene, first identified in *A. baumannii* in the early 1990s, is considered intrinsic to this species and is not inherently linked to carbapenem resistance. However, overexpression of this gene—often driven by upstream ISs such as *ISAba1* and, less commonly, *ISAba9* or *ISAba19*—can lead to low-level resistance [166,167,168,169]. Variants like OXA-65, OXA-66, and OXA-69, also part of the OXA-51 family, are commonly detected and possess carbapenemase activity [170]. Over the years, numerous other *bla*_OXA-51-like_ alleles such as *bla*_OXA-64_, *bla*_OXA-94_, *bla*_OXA-365_, *bla*_OXA-68_, *bla*_OXA-90_, *bla*_OXA-132_, *bla*_OXA-79_, *bla*_OXA-82_, *bla*_OXA-92_, and *bla*_OXA-131_ have emerged [171,172,173], along with the recently identified *bla*_OXA-1117_ and *bla*_OXA-1118_ [173]. Although this gene was once widely used as a diagnostic marker for *A. baumannii*, it is no longer considered sufficient for accurate species identification [169].

Until around 2010, OXA-58 was the dominant OXA-type carbapenemase in CRAB isolates, largely due to its higher enzymatic activity compared to intrinsic OXA-51. The *bla*_OXA-58-like_ gene is typically flanked by two *ISAba3-like* elements, a structure first described in France [170,174,175,176,177].

OXA-23, although initially detected earlier, gained prominence after 2010 and gradually replaced OXA-58 as the most common carbapenemase in CRAB due to its superior hydrolytic efficiency [178,179,180,181]. The *bla*_OXA-23-like_ gene remains dominant and is often found adjacent to *ISAba1*. In some endemic regions, strains co-harboring both *bla*_OXA-23-like_ and *bla*_OXA-58-like_ have been reported [182,183,184,185,186,187]. The OXA-24 variant—sharing approximately 60% identity with OXA-23—is more frequently seen in Spain, Portugal, and China [188,189,190].

Domingues et al. [48] reported a temporal shift in the prevalence of *bla*_OXA-like_ genes, with *bla*_OXA-40-like_ detected in 2005, subsequently replaced by *bla*_OXA-23_ between 2006 and 2019. This trend reflects the broader historical pattern in Portugal, where *bla*_OXA-58_ was predominant from 2002 to 2004, followed by *bla*_OXA-40_ (2002–2006), and later *bla*_OXA-23_ from 2006 onward. These shifts highlight the central role of Class D β-lactamases as the predominant carbapenemases in CRAB clinical isolates—a pattern also observed in reports from the United States and multiple Asian healthcare settings [46,48,189,191].

Mavroidi et al. [192] documented the emergence of *A. baumannii* isolates belonging to sequence type 3LST ST101, producing *bla*_OXA-23_ and classified as international clone II, which exhibited resistance to both tigecycline and colistin in a Greek hospital over a three-year span. According to the authors’ report [192], this represented the first reported occurrence of such isolates in Greece. The identification of two *bla*_OXA-23_-producing strains resistant to both agents is particularly alarming, highlighting the organism’s expanding ability to develop diverse antibiotic resistance mechanisms [17,192]. CRAB recruits intrinsic OXA-51-like enzymes as well as acquired forms such as OXA-23-like, OXA-40-like, and OXA-58-like for most strains worldwide [48].

The OXA-23 gene, a prominent member of the OXA family, is recognized as the most prevalent carbapenem resistance mechanism worldwide [45,48].

Other notable OXA-type carbapenemases include OXA-49 and OXA-73 (prevalent in China), and OXA-143 and OXA-231, primarily reported in Brazil [166].

*A. baumannii* isolates like CRAB strains display a complex resistance profile, with OXA-type carbapenemases representing the primary mechanism underlying resistance to carbapenems and other β-lactams. Additionally, target site modifications and efflux pump activity often contribute to resistance against key therapeutic agents, including polymyxins, cefiderocol, and other β-lactams [31].

### 4.2. Metallo-β-Lactamases (MBLs)

MBLs, particularly VIM enzymes, are the most frequently detected among CRAB strains. In endemic regions such as Greece, VIM-1 and VIM-4 are the most common [193,194,195,196], whereas VIM-2 predominates in South Korea [197,198]. The *bla*_VIM_ genes are consistently integrated within class 1 Ints [193,194]. Although rare, other variants like VIM-35 have also been identified [199]. Additional MBLs, including IMP-1 and SIM-1, have been reported in countries such as South Korea, Iran, and Morocco [131,200,201,202,203]. SPM-producing CRAB strains are also notably present in Morocco [203,204]. Recently, strains co-expressing VIM-2 and OXA-23 were identified in Tunisia [205].

Although VIM-producing CRAB strains have been predominant, the global emergence of NDM-1 and NDM-2 producers began after 2012 [206,207,208]. NDM-1, in particular, has spread extensively, leading to multiple severe outbreaks over the following years and remains widespread today [209,210]. *The bla*_NDM-1_ gene is typically preceded by the insertion sequence *ISAba125* [211,212]. Additional NDM variants have also been reported, including NDM-6 in Spain [213], NDM-9 in France (2023) [214], and NDM-5 in Thailand during the same year [215]. CRAB strains co-producing NDM-1 and OXA-58 have been identified [35,216], though the most commonly observed pattern is the coexistence of NDM-1 and OXA-23 in the same isolates [217,218,219,220,221].

In a study by Wang et al. [37], the resistome of 125 CRAB isolates was analyzed, revealing 47 unique ARGs. These genes conferred resistance to four major antibiotic classes of aminoglycosides, sulfonamides, tetracyclines, and β-lactams. A key finding was the presence of carbapenemases in all CRAB isolates.

Wang et al. [37] found that 26 ARGs were highly prevalent, being detected in more than half of the strains. The most common were *AdeFGH*, *bla*_OXA-66_, and *bla*_OXA-23_, respectively. Temporal patterns such as *bla*_OXA-51_, *bla*_OXA-217_, and *bla*_OXA-374_ were found only in 2019 isolates, while *bla*_NDM-1_ and *bla*_OXA-91_ were unique to 2023 isolates. No resistance genes of *mcr-1* (colistin) or *tet(X)* (tigecycline) were detected by Wang et al. [37]. However, two isolates from 2023 (e.g., 2023-AB023 and 2023-AB033) were identified by Wang et al. [37], harboring two different carbapenemase genes and a total of seven resistance genes, including *bla*_OXA-23_, *bla*_OXA-91_, and *bla*_NDM-1_.

### 4.3. Klebsiella pneumoniae Carbapenemases (KPCs)

KPC-type carbapenemases are relatively uncommon in CRAB isolates. The *bla*_KPC_ gene was first identified in *A. baumannii* strains from Puerto Rico in 2009, involving variants such as KPC-2, KPC-3, KPC-4, and KPC-10 [11,222]. However, its presence remained largely confined to that region in the following years [223]. Since then, only sporadic cases have been reported globally—for instance, a KPC-3-producing CRAB isolate in Portugal [224] and a recent case from Tunisia involving a strain co-producing OXA-23 and KPC-2 [225].

### 4.4. Sequence Types (STs)

The emergence of resistant Acinetobacter calcoaceticus–baumannii (Acb) strains has prompted widespread epidemiological research. MLST is now the standard tool due to its strong reproducibility, global consistency, and ability to trace evolutionary patterns. As an advantage, its use of standardized ST nomenclatures enables effective tracking of major clonal lineages [166,226,227]. In *A. baumannii*, MLST’s effectiveness is limited by the existence of two main schemes: Oxford and Pasteur. Though both classify similar strains, comparing their accuracy, resolution, and phylogenetic consistency remains necessary [226,228,229,230]. Molecular studies have identified major clonal lineages in *A. baumannii*, now known globally as International Clones (IC) I–III. In the Pasteur MLST scheme, these align with clonal complexes CC1 (ST1), CC2 (ST2), and CC3 (ST3). Other common sequence types include ST10, ST15, ST25, ST32, ST78, and ST79 [166,171,226,230,231,232,233]. The Oxford scheme provides higher resolution but is prone to recombination and technical issues, especially in the *gpi* gene, part of the capsule operon. As *gpi* affects capsule type and virulence, some studies recommend using this scheme for tracking capsular variation [226,234,235,236,237].

The updated epidemiology of *A. baumannii* sequence types, isolates, and genomes is available on the AcinetobacterPubMLST website (https://pubmlst.org/organisms/acinetobacter-baumannii, accessed on 27 June 2025) [238].

In detail, 2779 Pasteur STs and 3415 Oxford STs are available at https://pubmlst.org/bigsdb?db=pubmlst_abaumannii_seqdef&page=query (accessed on 27 June 2025). Also, 12570 genomes assigned to validated *Acinetobacter* spp. are available at https://pubmlst.org/bigsdb?db=pubmlst_abaumannii_isolates&page=query&genomes=1 (accessed on 27 June 2025) [238].

Before ST2 became dominant, ST1 was the most common sequence type [239]. The rise of ST2 has been linked to the spread of OXA-23-producing CRAB [178]. VIM-1 and VIM-4 enzymes have been associated with ST2 and ST1, respectively [166]. International Clones IC1 and IC2 correspond to ST1 and ST2, including their single-locus variants [240].

NDM-1-producing CRAB strains are typically assigned to ST25 [241]. Less frequently reported types include ST1407, ST164, and ST85 [242,243,244,245]. Other NDM variants have been linked to various sequence types: NDM-9 with ST25, NDM-5 with ST19, and NDM-2 with ST103 [214,246,247]. In recent years, a concerning trend has emerged with CRAB strains co-producing NDM-1 and OXA-23, most commonly associated with ST2 [211,221], though ST1 has also been identified in some cases [220].

## 5. Trends in Antimicrobial Treatment

A range of older and newly developed antimicrobials show potential as treatment options for CRAB infections. However, some of the recently introduced agents lack activity against these resistant strains [11,248,249].

### 5.1. Colistin—Polymyxins

Colistin (PubChem CID: 44144393, Figure 2a) is an old antimicrobial discovered in 1949 and classified as a polymyxin (specifically polymyxin E). Its clinical use was largely discontinued in the 1980s due to severe side effects, particularly nephrotoxicity [250].

The resurgence of CRAB and other MDR bacteria in recent years has led to the renewed use of colistin as a key therapeutic option [263].

Both colistin and polymyxin B have been employed to treat hospital-acquired pneumonia (HAP), ventilator-associated pneumonia (VAP), and bloodstream infections caused by CRAB [249]. However, colistin has been associated with a higher risk of nephrotoxicity compared to polymyxin B [264].

There are different reports from different studies. In an in vitro study performed by Vardakas et al. [265], colistin and other polymyxins have shown strong synergistic effects when combined with carbapenems, rifampicin, or vancomycin, which may contribute to improved survival outcomes [265]. Some clinical studies have found no significant difference in treatment failure rates between colistin monotherapy and combination therapies such as colistin–meropenem or colistin–rifampicin [266,267], while in an investigation conducted by Dickstein et al. [268], they studied 266 *A. baumannii* cases and found lower adjusted mortality in colistin-resistant infections compared to colistin-susceptible ones. Colistin-resistant patients had better baseline health and needed less ventilation [268]. Among colistin-resistant cases, colistin–meropenem therapy led to higher mortality than colistin alone [268]. Colistin resistance, based on broth microdilution (BMD) testing, was linked to improved outcomes, with monotherapy outperforming combination treatment [268].

A randomized controlled trial (RCT) comparing colistin with ampicillin–sulbactam for treating CRAB strains susceptible to ampicillin–sulbactam found no significant differences in mortality, clinical outcomes, or microbiological failure [269]. However, another study reported higher rates of all-cause mortality and microbiological failure with colistin. These findings suggest a possible advantage of ampicillin–sulbactam over polymyxins, though the overall quality of evidence remains low [270].

Colistin has demonstrated notable synergistic activity with levofloxacin in treating VAP caused by CRAB [271]. Given the emergence of colistin-resistant CRAB strains [192] and the risk of resistance development and treatment failure with colistin monotherapy [272,273], it is recommended that colistin be used as part of combination therapy for severe CRAB infections [274].

### 5.2. Ampicillin–Sulbactam

Ampicillin–sulbactam (PubChem CID: 18541918, Figure 2b) is another potential option for treating HAP/VAP caused by CRAB, although the supporting evidence is limited or conditional [249]. As aforementioned, it has shown a strong synergistic effect when combined with levofloxacin in managing CRAB-related VAP [271]. In the treatment of *A. baumannii* infections, the antibacterial activity of ampicillin–sulbactam is primarily attributed to its sulbactam component, with ampicillin functioning mainly as a carrier. Based on this understanding, the 2023 guidelines designated ampicillin–sulbactam (6–9 g of sulbactam daily), in combination with at least one other agent, as the preferred therapy for CRAB [275]. This recommendation applied regardless of in vitro susceptibility profiles, due to concerns over potential PBP saturation and testing inaccuracies. A significant shift occurred in 2024, when sulbactam–durlobactam, administered with a carbapenem, became the first-line treatment [275]. Ampicillin–sulbactam was subsequently reclassified as an alternative therapy, limited to the higher sulbactam dose of 9 g per day. Polymyxin B, minocycline, and cefiderocol are recommended as combination partners in such regimens. Differences between ESCMID and Infectious Diseases Society of America (IDSA) guidelines likely reflect the regional timing of sulbactam–durlobactam’s approval in 2023 across the United States and Europe [275]. Xacduro, a fixed-dose sulbactam–durlobactam therapy approved in the U.S., treats HAP and VAP caused by the *A. baumannii–calcoaceticus* complex. Sulbactam acts as the antibacterial agent, while durlobactam, a DBO beta-lactamase (classes A, C, and D) inhibitor, shields it from enzymatic breakdown. Though durlobactam lacks direct killing power, it boosts sulbactam’s efficacy. In vitro, the combo rivals colistin and outperforms several other antibiotics against CRAB. Clinically, it improves outcomes with fewer deaths and less kidney toxicity than colistin. Since its effect without carbapenems remains uncertain, the IDSA advises using it alongside imipenem–cilastatin or meropenem [115,276,277,278,279,280].

### 5.3. Tigecycline

Tigecycline (PubChem CID: 54686904, Figure 2c), a glycylcycline and derivative of minocycline, reaches high concentrations in the lungs, skin, soft tissues, and bones [281]. It demonstrates strong activity against CRAB strains; however, exposure to suboptimal tigecycline levels may promote resistance development [282].

Compared to colistin in treating CRAB pneumonia, tigecycline monotherapy has been associated with higher mortality and lower clinical response [283,284,285]. Conversely, one study reported better survival rates with tigecycline than colistin [286], though overall evidence remains inconclusive [249].

When assessed against ampicillin–sulbactam, tigecycline-based therapies showed lower clinical and microbiological failure rates [287]. In contrast, another study found significantly lower 28-day mortality with cefoperazone–sulbactam compared to tigecycline [288]. However, the evidence for the superiority of sulbactam-based regimens over tigecycline is still of low certainty [249].

### 5.4. Fosfomycin

Fosfomycin (PubChem CID: 446987, Figure 2d) is an older antibiotic that has been reintroduced, primarily for treating uncomplicated urinary tract infections (UTIs) caused by MDR uropathogens [87,96,289,290,291]. In the context of CRAB infections, it has been used in combination therapies for VAP and bacteremia. However, its clinical effectiveness in these settings requires further confirmation through larger, well-designed studies [292,293].

### 5.5. Plazomicin

Plazomicin (PubChem CID: 42613186, Figure 2e) is a synthetic aminoglycoside approved in 2018 for the treatment of complicated urinary tract infections (cUTIs) and pyelonephritis [248]. Early studies suggest it has some in vitro activity against CRAB [294]. Despite its enhanced potency compared to other aminoglycosides, plazomicin is not regarded as a first-line treatment for CRAB infections [249,295].

### 5.6. Eravacycline

Eravacycline (PubChem CID: 54726192, Figure 2f) is a fluorocycline that is two to eight times more potent than tigecycline against CRAB. While some clinical evidence supports its effectiveness, eravacycline is currently considered a last-resort treatment, and further research is needed to validate its clinical utility [249,296].

### 5.7. Cefiderocol

Cefiderocol (PubChem CID: 77843966, Figure 2g) is a novel siderophore cephalosporin approved by the FDA for the treatment of cUTIs in 2019 and VAP in 2020 [297]. It also received marketing authorization from the European Medicines Agency (EMA) for treating infections caused by aerobic MDR Gram-negative bacteria in adults with limited treatment options [298]. Cefiderocol utilizes siderophore-mediated transport to penetrate the bacterial outer membrane and accumulate in the periplasmic space, enabling it to inhibit a broad range of MDR bacteria regardless of the resistance mechanism [299].

Against CRAB, cefiderocol shows a minimum inhibitory concentration (MIC) of ≤2 μg/mL and has demonstrated a 70% survival rate in critically ill patients with bacteremia or VAP in one study [300]. However, the CREDIBLE-CR randomized controlled trial comparing cefiderocol with best available therapy for carbapenem-resistant Gram-negative infections reported a higher 28-day mortality rate among CRAB patients treated with cefiderocol (49%) compared to those receiving other treatments (18%) [301]. While cefiderocol has been used in managing severe CRAB outbreaks [210], emerging resistance poses a growing concern, with some studies linking cefiderocol resistance to clinical failure. As a result, cefiderocol monotherapy for CRAB is generally not recommended [302]. Overall, data on its efficacy against CRAB remain limited, and further research is required [249].

### 5.8. Temocillin

Temocillin (PubChem CID: 171758, Figure 2h) is a 6-α-methoxy derivative of ticarcillin introduced in the UK during the 1980s. While it exhibits activity against various Enterobacterales, it has no efficacy against *A. baumannii* or CRAB [248,303].

### 5.9. Ceftolozane–Tazobactam

Ceftolozane–tazobactam (PubChem CID: 172973390, Figure 2i) received FDA approval in 2014 for the treatment of cUTIs and intra-abdominal infections (IAIs), with its use later extended to VAP in 2019 [248]. In 2022, approval was further expanded to include pediatric patients (from birth to under 18 years) for cIAIs and cUTIs [304]. However, it shows limited in vivo activity against CRAB [305,306].

### 5.10. Imipenem/Cilastatin–Relebactam

Imipenem/cilastatin–relebactam (PubChem CID: --) was approved by the FDA in 2019 for the treatment of cUTIs and IAIs, and in 2020, its approval was extended to include VAP. It has also been authorized by the EMA [248]. Relebactam functions by inhibiting class A and C β-lactamases [307]. However, this combination is not considered effective against CRAB, as the addition of relebactam does not enhance imipenem’s in vitro activity against these strains [308,309].

### 5.11. Meropenem–Vaborbactam

Vaborbactam is a cyclic boronate β-lactamase inhibitor. When combined with meropenem, it is effective against KPC-producing multidrug-resistant Gram-negative bacteria and shows notable activity against OXA-48 producers. This combination, meropenem–vaborbactam (PubChem CID: 86298703, Figure 2j), is approved for the treatment of cUTIs, IAIs, and VAP [310,311]. However, it is not considered a viable option for CRAB, as vaborbactam does not enhance meropenem’s in vitro activity against these strains [308,309].

### 5.12. Ceftazidime–Avibactam

Avibactam is a non-β-lactam β-lactamase inhibitor with in vitro activity against Ambler class A and C β-lactamases and partial activity against certain OXA-type enzymes classified under Ambler class D. It was patented in 2011 [312]. Several RCTs have evaluated the safety and effectiveness of the ceftazidime–avibactam combination for treating cUTIs and cIAIs [313].

Ceftazidime–avibactam (PubChem CID: 90643431, Figure 2k) was approved by the FDA in 2015 for the treatment of cUTIs and, in combination with metronidazole, for cIAIs [314]. Its dosing was later reassessed for use in critically ill patients [315]. In 2018, the FDA extended its approval to include HAP/VAP, based on results from the pivotal Phase III REPROVE trial [316]. However, data on its efficacy against CRAB are limited, and it is therefore not routinely recommended for these infections [249,317].

### 5.13. Aztreonam–Avibactam

Aztreonam–avibactam (PubChem CID: --) is a combination antimicrobial agent effective against MDR bacteria producing MBLs [318]. It was approved by the EMA in 2024 for the treatment of infections such as cIAIs, cUTIs, and HAP/VAP [319]. In February 2025, the FDA approved it for treating cIAIs in adults with limited therapeutic options [320]. In the context of CRAB, aztreonam–avibactam is particularly effective against strains producing MBLs such as VIM or NDM [319,321,322].

## 6. Guidelines for the Treatment of CRAB Infections

According to the latest guidelines from the European Society of Clinical Microbiology and Infectious Diseases (ESCMID), ampicillin–sulbactam is conditionally recommended for patients with HAP/VAP caused by sulbactam-susceptible CRAB. Alternatives include polymyxins or high-dose tigecycline, provided in vitro activity is confirmed; however, the quality of evidence is low. For CRAB strains resistant to sulbactam, polymyxins or high-dose tigecycline may be considered if susceptibility is demonstrated, though no formal recommendation is provided. ESCMID also conditionally advises against the use of cefiderocol for CRAB infections, citing low-quality evidence for this guidance [249,323].

For patients with severe, high-risk CRAB infections, ESCMID conditionally recommends combination therapy using two antimicrobials with confirmed in vitro activity—such as polymyxins, aminoglycosides, tigecycline, or sulbactam-based regimens. However, this recommendation is based on low-quality evidence. Combination treatments involving polymyxin–meropenem or polymyxin–rifampin are strongly discouraged, supported by high-quality evidence. In cases where CRAB isolates have a meropenem MIC ≤ 8 mg/L, a carbapenem-based combination therapy using high-dose, extended-infusion meropenem is considered good clinical practice, although this guidance is based on expert opinion [249,274,275].

The IDSA recommends ampicillin–sulbactam as the first-line treatment for CRAB isolates susceptible to sulbactam. Alternative options include tetracyclines (such as eravacycline or minocycline), tigecycline, polymyxins, and cefiderocol. For sulbactam-resistant CRAB, IDSA provides no formal recommendation, but the use of high-dose ampicillin–sulbactam in combination with a second active agent may be considered [324,325,326].

For severe CRAB infections, IDSA strongly supports combination therapy using at least two active antimicrobials. High-dose ampicillin–sulbactam should serve as the core agent, combined with another drug such as a tetracycline, polymyxin, extended-infusion meropenem, or cefiderocol. Fosfomycin and rifampin are not recommended as part of combination regimens. Similarly, polymyxin–meropenem combinations should be avoided unless a third agent is included [274,327].

## 7. Conclusions

Pan-genomic analyses have revealed that *A. baumannii* has an open pan-genome, containing a wide array of virulence factors. Most of its virulence and ARGs are disseminated via MGEs, including Tns, ISs, Ints, and bacteriophages (Φs)—often transferred through plasmids via HGT. As a result, *A. baumannii* harbors a robust arsenal of resistance and virulence mechanisms, making CRAB infections a serious public health concern [12,21,24].

Understanding the specific mechanisms of antimicrobial resistance is crucial for selecting effective treatment options. Potential therapies include older agents such as polymyxins, ampicillin–sulbactam, high-dose carbapenems, tigecycline, and minocycline, as well as newer drugs like eravacycline, cefiderocol, and aztreonam–avibactam.

## Figures and Tables

**Figure 2:**
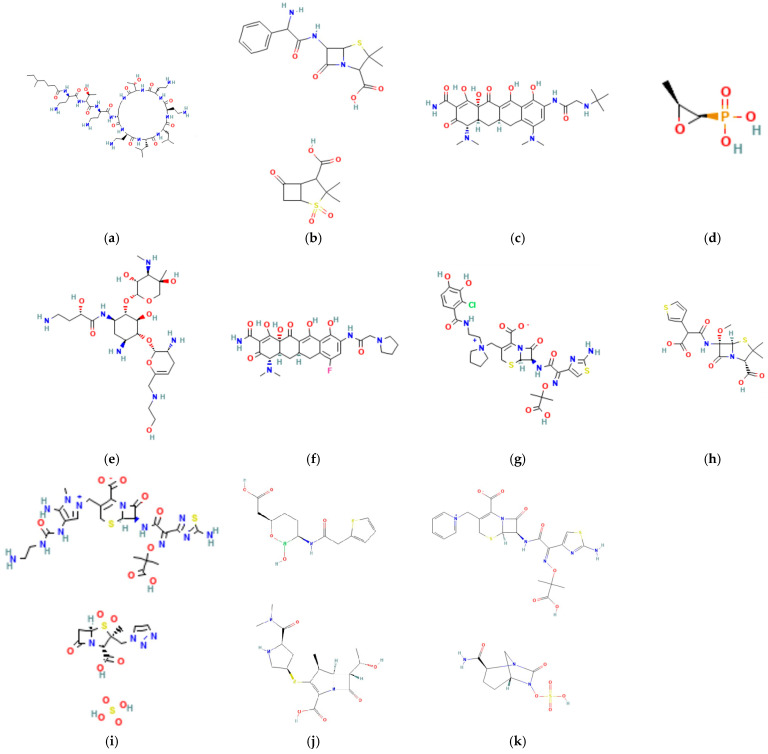
Two-dimensional structures of antimicrobial agents including (**a**) colistin (polymyxin class) (2D structure of colistin; C_52_H_98_N_16_O_13_; https://pubchem.ncbi.nlm.nih.gov/compound/44144393) [251,252]; (**b**) ampicillin–sulbactam (2D structure of ampicillin–sulbactam; C_25_H_31_N_3_O_9_S_2_; https://pubchem.ncbi.nlm.nih.gov/compound/18541918) [253] (penicillins class-β-lactamase inhibitor) [252], (**c**) tigecycline (2D structure of tigecycline; C_29_H_39_N_5_O_8_; https://pubchem.ncbi.nlm.nih.gov/compound/54686904) [254] (glycylcyclines class) [252], (**d**) Fosfomycin (2D structure of fosfomycin; C_3_H_7_O_4_P; https://pubchem.ncbi.nlm.nih.gov/compound/446987) [255] (organic phosphonic acids class) [252], (**e**) plazomicin (2D structure of plazomicin; C_25_H_48_N_6_O_10_; https://pubchem.ncbi.nlm.nih.gov/compound/42613186) [256] (aminoglycosides class) [252], (**f**) eravacycline (2D structure of eravacycline; C_27_H_31_FN_4_O_8_; https://pubchem.ncbi.nlm.nih.gov/compound/54726192) [257] (tetracyclines class) [252], (**g**) cefiderocol (2D structure of cefiderocol; C_30_H_34_ClN_7_O_10_S_2_; https://pubchem.ncbi.nlm.nih.gov/compound/77843966) [258] (cephalosporins class) [252], (**h**) temocillin (2D structure of temocillin; C_16_H_18_N_2_O_7_S_2_; https://pubchem.ncbi.nlm.nih.gov/compound/171758) [259] (carboxylic acids class) [252], (**i**) ceftolozane–tazobactam (2D structure of ceftolozane–tazobactam; C_33_H_44_N_16_O_17_S_4_; https://pubchem.ncbi.nlm.nih.gov/compound/172973390) [260] (cephalosporins class-β-lactamase inhibitor) [252], (**j**) meropenem–vaborbactam (2D structure of meropenem–vaborbactam; C_29_H_41_BN_4_O_10_S_2_; https://pubchem.ncbi.nlm.nih.gov/compound/86298703) [261] (carbapenems class-β-lactamase inhibitor) [252], and (**k**) ceftazidime–avibactam (2D structure of ceftazidime–avibactam; C_29_H_33_N_9_O_13_S_3_; https://pubchem.ncbi.nlm.nih.gov/compound/90643431) [262] (cephalosporins class-non-β-lactam β-lactamase inhibitor) [252] against CRAB isolates (https://www.ncbi.nlm.nih.gov/; https://go.drugbank.com//drugs; accessed on 27 June 2025).

## Data Availability

No new data were created or analyzed in this study. Data sharing is not applicable to this article.

## References

[1] Cogliati Dezza F., Covino S., Petrucci F., Sacco F., Viscido A., Gavaruzzi F., Ceccarelli G., Raponi G., Borrazzo C., Alessandri F. (2023). Risk factors for carbapenem-resistant *Acinetobacter baumannii* (CRAB) bloodstream infections and related mortality in critically ill patients with CRAB colonization. JAC-Antimicrob. Resist..

[2] Doughty E.L., Liu H., Moran R.A., Hua X., Ba X., Guo F., Chen X., Zhang L., Holmes M., van Schaik W. (2023). Endemicity and diversification of carbapenem-resistant *Acinetobacter baumannii* in an intensive care unit. Lancet Reg. Health. West. Pac..

[3] Lemos E.V., de la Hoz F.P., Alvis N., Einarson T.R., Quevedo E., Castaneda C., Leon Y., Amado C., Canon O., Kawai K. (2014). Impact of carbapenem resistance on clinical and economic outcomes among patients with *Acinetobacter baumannii* infection in Colombia. Clin. Microbiol. Infect. Off. Publ. Eur. Soc. Clin. Microbiol. Infect. Dis..

[4] Villegas M.V., Hartstein A.I. (2003). Acinetobacter outbreaks, 1977–2000. Infect. Control Hosp. Epidemiol..

[5] Routsi C., Pratikaki M., Platsouka E., Sotiropoulou C., Nanas S., Markaki V., Vrettou C., Paniara O., Giamarellou H., Roussos C. (2010). Carbapenem-resistant versus carbapenem-susceptible *Acinetobacter baumannii* bacteremia in a Greek intensive care unit: Risk factors, clinical features and outcomes. Infection.

[6] Sati H., Carrara E., Savoldi A., Hansen P., Garlasco J., Campagnaro E., Boccia S., Castillo-Polo J.A., Magrini E., Garcia-Vello P. (2025). The WHO Bacterial Priority Pathogens List 2024: A prioritisation study to guide research, development, and public health strategies against antimicrobial resistance. Lancet Infect. Dis..

[7] Karampatakis T., Antachopoulos C., Tsakris A., Roilides E. (2017). Molecular epidemiology of carbapenem-resistant *Acinetobacter baumannii* in Greece: An extended review (2000–2015). Future Microbiol..

[8] European Centre for Disease Prevention and Control (2023). Surveillance Atlas of Infectious Diseases. https://atlas.ecdc.europa.eu/public/index.aspx?Dataset=27&HealthTopic=4.

[9] Magiorakos A.P., Srinivasan A., Carey R.B., Carmeli Y., Falagas M.E., Giske C.G., Harbarth S., Hindler J.F., Kahlmeter G., Olsson-Liljequist B. (2012). Multidrug-resistant, extensively drug-resistant and pandrug-resistant bacteria: An international expert proposal for interim standard definitions for acquired resistance. Clin. Microbiol. Infect. Off. Publ. Eur. Soc. Clin. Microbiol. Infect. Dis..

[10] Golpasand T., Keshvari M., Behzadi P. (2024). Distribution of chaperone-usher fimbriae and curli fimbriae among uropathogenic *Escherichia coli*. BMC Microbiol..

[11] Karampatakis T., Tsergouli K., Behzadi P. (2023). Carbapenem-Resistant Klebsiella pneumoniae: Virulence Factors, Molecular Epidemiology and Latest Updates in Treatment Options. Antibiotics.

[12] Sarshar M., Behzadi P., Scribano D., Palamara A.T., Ambrosi C. (2021). *Acinetobacter baumannii*: An Ancient Commensal with Weapons of a Pathogen. Pathogens.

[13] Algammal A.M., Behzadi P. (2024). Antimicrobial Resistance: A Global Public Health Concern that Needs Perspective Combating Strategies and New Talented Antibiotics. Discov. Med..

[14] Viehman J.A., Nguyen M.H., Doi Y. (2014). Treatment options for carbapenem-resistant and extensively drug-resistant *Acinetobacter baumannii* infections. Drugs.

[15] Karakonstantis S., Gikas A., Astrinaki E., Kritsotakis E.I. (2020). Excess mortality due to pandrug-resistant *Acinetobacter baumannii* infections in hospitalized patients. J. Hosp. Infect..

[16] Kritsotakis E.I., Lagoutari D., Michailellis E., Georgakakis I., Gikas A. (2022). Burden of multidrug and extensively drug-resistant ESKAPEE pathogens in a secondary hospital care setting in Greece. Epidemiol. Infect..

[17] Papadopoulou M., Deliolanis I., Polemis M., Vatopoulos A., Psichogiou M., Giakkoupi P. (2024). Characteristics of the Genetic Spread of Carbapenem-Resistant *Acinetobacter baumannii* in a Tertiary Greek Hospital. Genes.

[18] Wu H.J., Xiao Z.G., Lv X.J., Huang H.T., Liao C., Hui C.Y., Xu Y., Li H.F. (2023). Drug-resistant *Acinetobacter baumannii*: From molecular mechanisms to potential therapeutics (Review). Exp. Ther. Med..

[19] Medini D., Donati C., Tettelin H., Masignani V., Rappuoli R. (2005). The microbial pan-genome. Curr. Opin. Genet. Dev..

[20] Urhan A., Abeel T. (2021). A comparative study of pan-genome methods for microbial organisms: *Acinetobacter baumannii* pan-genome reveals structural variation in antimicrobial resistance-carrying plasmids. Microb. Genom..

[21] Karampatakis T., Tsergouli K., Behzadi P. (2024). Pan-Genome Plasticity and Virulence Factors: A Natural Treasure Trove for *Acinetobacter baumannii*. Antibiotics.

[22] Tantoso E., Eisenhaber B., Kirsch M., Shitov V., Zhao Z., Eisenhaber F. (2022). To kill or to be killed: Pangenome analysis of *Escherichia coli* strains reveals a tailocin specific for pandemic ST131. BMC Biol..

[23] Mira A., Martin-Cuadrado A.B., D’Auria G., Rodriguez-Valera F. (2010). The bacterial pan-genome:a new paradigm in microbiology. Int. Microbiol. Off. J. Span. Soc. Microbiol..

[24] Karampatakis T., Tsergouli K., Behzadi P. (2025). Carbapenem-Resistant *Pseudomonas aeruginosa*’s Resistome: Pan-Genomic Plasticity, the Impact of Transposable Elements and Jumping Genes. Antibiotics.

[25] Lean S.S., Yeo C.C. (2017). Small, Enigmatic Plasmids of the Nosocomial Pathogen, *Acinetobacter baumannii*: Good, Bad, Who Knows?. Front. Microbiol..

[26] Noel H.R., Petrey J.R., Palmer L.D. (2022). Mobile genetic elements in Acinetobacter antibiotic-resistance acquisition and dissemination. Ann. N. Y. Acad. Sci..

[27] Costa A.R., Monteiro R., Azeredo J. (2018). Genomic analysis of *Acinetobacter baumannii* prophages reveals remarkable diversity and suggests profound impact on bacterial virulence and fitness. Sci. Rep..

[28] Behzadi P., Ambrosi C., Scribano D., Zanetti S., Sarshar M., Gajdacs M., Donadu M.G. (2022). Editorial: Current perspectives on *Pseudomonas aeruginosa*: Epidemiology, virulence and contemporary strategies to combat multidrug-resistant (MDR) pathogens. Front. Microbiol..

[29] Hamidian M., Hawkey J., Wick R., Holt K.E., Hall R.M. (2019). Evolution of a clade of *Acinetobacter baumannii* global clone 1, lineage 1 via acquisition of carbapenem- and aminoglycoside-resistance genes and dispersion of ISAba1. Microb. Genom..

[30] Partridge S.R., Kwong S.M., Firth N., Jensen S.O. (2018). Mobile Genetic Elements Associated with Antimicrobial Resistance. Clin. Microbiol. Rev..

[31] Castanheira M., Mendes R.E., Gales A.C. (2023). Global epidemiology and mechanisms of resistance of *Acinetobacter baumannii*-calcoaceticus complex. Clin. Infect. Dis..

[32] Algammal A., Hetta H.F., Mabrok M., Behzadi P. (2023). Emerging multidrug-resistant bacterial pathogens “superbugs”: A rising public health threat. Front. Microbiol..

[33] Matos A.P., Cayô R., Almeida L.G., Streling A.P., Nodari C.S., Martins W.M., Narciso A.C., Silva R.M., Vasconcelos A.T., Gales A.C. (2019). Genetic characterization of plasmid-borne bla OXA-58 in distinct Acinetobacter species. Msphere.

[34] Alattraqchi A., Rani F., Rahman N., Ismail S., Cleary D., Clarke S., Yeo C. (2020). Complete genome sequencing of *Acinetobacter baumannii* AC1633 andAcinetobacter nosocomialisAC1530 unveils a large multidrug resistant plasmid encoding the NDM-1 and OXA-58 carbapenemases. Msphere.

[35] Liu H., Moran R.A., Chen Y., Doughty E.L., Hua X., Jiang Y., Xu Q., Zhang L., Blair J.M., McNally A. (2021). Transferable *Acinetobacter baumannii* plasmid pDETAB2 encodes OXA-58 and NDM-1 and represents a new class of antibiotic resistance plasmids. J. Antimicrob. Chemother..

[36] Ramoul A., Loucif L., Bakour S., Amiri S., Dekhil M., Rolain J.-M. (2016). Co-occurrence of blaNDM-1 with blaOXA-23 or blaOXA-58 in clinical multidrug-resistant *Acinetobacter baumannii* isolates in Algeria. J. Glob. Antimicrob. Resist..

[37] Wang X., Zhao B., Zhou Y., Zhang Y., Xu T., Zhuang Y., Chen M., Hao L., Shen Y., Feng J. (2025). Genomic insights of the co-existence of blaOXA-23, blaOXA-91, blaNDM-1 harboring carbapenem-resistant *Acinetobacter baumannii* isolates from the intensive care units environment in Shanghai. J. Glob. Antimicrob. Resist..

[38] Shadan A., Pathak A., Ma Y., Pathania R., Singh R.P. (2023). Deciphering the virulence factors, regulation, and immune response to *Acinetobacter baumannii* infection. Front. Cell. Infect. Microbiol..

[39] Wachino J.I., Jin W., Kimura K., Arakawa Y. (2019). Intercellular Transfer of Chromosomal Antimicrobial Resistance Genes between *Acinetobacter baumannii* Strains Mediated by Prophages. Antimicrob. Agents Chemother..

[40] Brovedan M.A., Cameranesi M.M., Limansky A.S., Moran-Barrio J., Marchiaro P., Repizo G.D. (2020). What do we know about plasmids carried by members of the Acinetobacter genus?. World J. Microbiol. Biotechnol..

[41] Geisinger E., Huo W., Hernandez-Bird J., Isberg R.R. (2019). *Acinetobacter baumannii*: Envelope determinants that control drug resistance, virulence, and surface variability. Annu. Rev. Microbiol..

[42] Wyres K.L., Cahill S.M., Holt K.E., Hall R.M., Kenyon J.J. (2019). Identification of *Acinetobacter baumannii* loci for capsular polysaccharide (KL) and lipooligosaccharide outer core (OCL) synthesis in genome assemblies using curated reference databases compatible with Kaptive. Microb. Genom..

[43] Russo T.A., Luke N.R., Beanan J.M., Olson R., Sauberan S.L., MacDonald U., Schultz L.W., Umland T.C., Campagnari A.A. (2010). The K1 capsular polysaccharide of *Acinetobacter baumannii* strain 307-0294 is a major virulence factor. Infect. Immun..

[44] Pérez-Varela M., Tierney A.R., Dawson E., Hutcheson A.R., Tipton K.A., Anderson S.E., Haldopoulos M.E., Song S., Tomlinson B.R., Shaw L.N. (2022). Stochastic activation of a family of TetR type transcriptional regulators controls phenotypic heterogeneity in *Acinetobacter baumannii*. PNAS Nexus.

[45] Nguyen M., Joshi S. (2021). Carbapenem resistance in *Acinetobacter baumannii*, and their importance in hospital-acquired infections: A scientific review. J. Appl. Microbiol..

[46] Silva L., Grosso F., Rodrigues C., Ksiezarek M., Ramos H., Peixe L. (2021). The success of particular *Acinetobacter baumannii* clones: Accumulating resistance and virulence inside a sugary shield. J. Antimicrob. Chemother..

[47] Oliveira H., Costa A.R., Ferreira A., Konstantinides N., Santos S.B., Boon M., Noben J.-P., Lavigne R., Azeredo J. (2019). Functional analysis and antivirulence properties of a new depolymerase from a myovirus that infects *Acinetobacter baumannii* capsule K45. J. Virol..

[48] Domingues R., Oliveira R., Silva S., Araújo D., Almeida C., Cho G.-S., Franz C.M., Saavedra M.J., Azeredo J., Oliveira H. (2024). Molecular detection of carbapenemases in *Acinetobacter baumannii* strains of Portugal and association with sequence types, capsular types, and virulence. Clin. Ther..

[49] Gao Y., Li H., Chen H., Zhang J., Wang R., Wang Z., Wang H. (2022). Origin, phylogeny, and transmission of the epidemic clone ST208 of carbapenem-resistant *Acinetobacter baumannii* on a global scale. Microbiol. Spectr..

[50] Hamidian M., Nigro S.J. (2019). Emergence, molecular mechanisms and global spread of carbapenem-resistant *Acinetobacter baumannii*. Microb. Genom..

[51] Luo Y.-C., Hsieh Y.-C., Wu J.-W., Quyen T.L.T., Chen Y.-Y., Liao W.-C., Li S.-W., Wang S.-H., Pan Y.-J. (2022). Exploring the association between capsular types, sequence types, and carbapenemase genes in *Acinetobacter baumannii*. Int. J. Antimicrob. Agents.

[52] Kikuchi-Ueda T., Ubagai T., Kamoshida G., Nakano R., Nakano A., Ono Y. (2021). *Acinetobacter baumannii* LOS Regulate the Expression of Inflammatory Cytokine Genes and Proteins in Human Mast Cells. Pathogens.

[53] Behzadi P., Chandran D., Chakraborty C., Bhattacharya M., Saikumar G., Dhama K., Chakraborty A., Mukherjee S., Sarshar M. (2025). The dual role of toll-like receptors in COVID-19: Balancing protective immunity and immunopathogenesis. Int. J. Biol. Macromol..

[54] Behzadi P., Kim C.H., Pawlak E.A., Algammal A. (2023). Editorial: The innate and adaptive immune system in human urinary system. Front. Immunol..

[55] Gudueva E., Chemisova O. (2023). Pathogenicity factors of *Acinetobacter baumannii*. Med. Her. South Russ..

[56] Luke N.R., Sauberan S.L., Russo T.A., Beanan J.M., Olson R., Loehfelm T.W., Cox A.D., St. Michael F., Vinogradov E.V., Campagnari A.A. (2010). Identification and characterization of a glycosyltransferase involved in *Acinetobacter baumannii* lipopolysaccharide core biosynthesis. Infect. Immun..

[57] Tiku V., Kew C., Kofoed E.M., Peng Y., Dikic I., Tan M.-W. (2022). *Acinetobacter baumannii* secretes a bioactive lipid that triggers inflammatory signaling and cell death. Front. Microbiol..

[58] Talyansky Y., Nielsen T.B., Yan J., Carlino-Macdonald U., Di Venanzio G., Chakravorty S., Ulhaq A., Feldman M.F., Russo T.A., Vinogradov E. (2021). Capsule carbohydrate structure determines virulence in *Acinetobacter baumannii*. PLoS Pathog..

[59] Lucidi M., Visaggio D., Migliaccio A., Capecchi G., Visca P., Imperi F., Zarrilli R. (2024). Pathogenicity and virulence of *Acinetobacter baumannii*: Factors contributing to the fitness in healthcare settings and the infected host. Virulence.

[60] Boll J.M., Tucker A.T., Klein D.R., Beltran A.M., Brodbelt J.S., Davies B.W., Trent M.S. (2015). Reinforcing lipid A acylation on the cell surface of *Acinetobacter baumannii* promotes cationic antimicrobial peptide resistance and desiccation survival. MBio.

[61] Herrera C.M., Voss B.J., Trent M.S. (2021). Homeoviscous adaptation of the *Acinetobacter baumannii* outer membrane: Alteration of lipooligosaccharide structure during cold stress. mBio.

[62] Thacharodi A., Vithlani A., Hassan S., Alqahtani A., Pugazhendhi A. (2024). Carbapenem-resistant *Acinetobacter baumannii* raises global alarm for new antibiotic regimens. Iscience.

[63] Roy S., Junghare V., Dutta S., Hazra S., Basu S. (2022). Differential binding of carbapenems with the AdeABC efflux pump and modulation of the expression of AdeB linked to novel mutations within two-component system AdeRS in carbapenem-resistant *Acinetobacter baumannii*. MSystems.

[64] Thacharodi A., Lamont I.L. (2022). Aminoglycoside resistance in *Pseudomonas aeruginosa*: The contribution of the MexXY-OprM efflux pump varies between isolates. J. Med. Microbiol..

[65] Yoon E.-J., Nait Chabane Y., Goussard S., Snesrud E., Courvalin P., Dé E., Grillot-Courvalin C. (2015). Contribution of resistance-nodulation-cell division efflux systems to antibiotic resistance and biofilm formation in *Acinetobacter baumannii*. mBio.

[66] Dou Q., Zou M., Li J., Wang H., Hu Y., Liu W.e. (2017). AdeABC efflux pump and resistance of *Acinetobacter baumannii* against carbapenem. Zhong Nan Da Xue Xue Bao. Yi Xue Ban= J. Cent. South. Univ. Med. Sci..

[67] Thacharodi A., Lamont I.L. (2023). Gene–gene interactions reduce aminoglycoside susceptibility of *Pseudomonas aeruginosa* through efflux pump-dependent and-independent mechanisms. Antibiotics.

[68] Zhu L.-J., Pan Y., Gao C.-Y., Hou P.-F. (2020). Distribution of carbapenemases and efflux pump in carbapenem-resistance *Acinetobacter baumannii*. Ann. Clin. Lab. Sci..

[69] Dolma K.G., Khati R., Paul A.K., Rahmatullah M., de Lourdes Pereira M., Wilairatana P., Khandelwal B., Gupta C., Gautam D., Gupta M. (2022). Virulence characteristics and emerging therapies for biofilm-forming *Acinetobacter baumannii*: A review. Biology.

[70] Dahdouh E., Gómez-Gil R., Pacho S., Mingorance J., Daoud Z., Suárez M. (2017). Clonality, virulence determinants, and profiles of resistance of clinical *Acinetobacter baumannii* isolates obtained from a Spanish hospital. PLoS ONE.

[71] Chapartegui-González I., Lázaro-Díez M., Bravo Z., Navas J., Icardo J.M., Ramos-Vivas J. (2018). *Acinetobacter baumannii* maintains its virulence after long-time starvation. PLoS ONE.

[72] Choi C.H., Lee J.S., Lee Y.C., Park T.I., Lee J.C. (2008). *Acinetobacter baumannii* invades epithelial cells and outer membrane protein A mediates interactions with epithelial cells. BMC Microbiol..

[73] Smani Y., Fàbrega A., Roca I., Sánchez-Encinales V., Vila J., Pachón J. (2014). Role of OmpA in the multidrug resistance phenotype of *Acinetobacter baumannii*. Antimicrob. Agents Chemother..

[74] Sarshar M., Scribano D., Behzadi P., Masotti A., Ambrosi C. (2022). Outer membrane vesicles are the powerful cell-to-cell communication vehicles that allow bacteria to monitor extracellular milieu. ExRNA.

[75] Gaddy J.A., Actis L.A. (2009). Regulation of *Acinetobacter baumannii* biofilm formation. Future Microbiol..

[76] Fattahian Y., Rasooli I., Gargari S.L.M., Rahbar M.R., Astaneh S.D.A., Amani J. (2011). Protection against *Acinetobacter baumannii* infection via its functional deprivation of biofilm associated protein (Bap). Microb. Pathog..

[77] López C., Ayala J.A., Bonomo R.A., González L.J., Vila A.J. (2019). Protein determinants of dissemination and host specificity of metallo-β-lactamases. Nat. Commun..

[78] Capodimonte L., Meireles F.T.P., Bahr G., Bonomo R.A., Dal Peraro M., López C., Vila A.J. (2025). OXA β-lactamases from *Acinetobacter* spp. are membrane bound and secreted into outer membrane vesicles. mBio.

[79] Pereira I.L., Hartwig D.D. (2025). Unveiling the role of adhesin proteins in controlling *Acinetobacter baumannii* infections: A systematic review. Infect. Immun..

[80] Brossard K.A., Campagnari A.A. (2012). The *Acinetobacter baumannii* biofilm-associated protein plays a role in adherence to human epithelial cells. Infect. Immun..

[81] Smani Y., McConnell M.J., Pachón J. (2012). Role of fibronectin in the adhesion of *Acinetobacter baumannii* to host cells. PLoS ONE.

[82] Geng J., Henry N. (2011). Short time-scale bacterial adhesion dynamics. Bacterial Adhesion: Chemistry, Biology and Physics.

[83] Ishikawa M., Nakatani H., Hori K. (2012). AtaA, a new member of the trimeric autotransporter adhesins from Acinetobacter sp. Tol 5 mediating high adhesiveness to various abiotic surfaces. PLoS ONE.

[84] Berne C., Ducret A., Hardy G.G., Brun Y.V. (2015). Adhesins involved in attachment to abiotic surfaces by Gram-negative bacteria. Microbial biofilms.

[85] Loehfelm T.W., Luke N.R., Campagnari A.A. (2008). Identification and characterization of an *Acinetobacter baumannii* biofilm-associated protein. J. Bacteriol..

[86] Suh J.W., Park S.M., Ju Y.K., Yang K.S., Kim J.Y., Kim S.B., Sohn J.W., Yoon Y.K. (2024). Clinical and molecular predictors of mortality in patients with carbapenem-resistant *Acinetobacter baumannii* bacteremia: A retrospective cohort study. J. Microbiol. Immunol. Infect..

[87] Behzadi P., Najafi A., Behzadi E., Ranjbar R. (2016). Microarray long oligo probe designing for *Escherichia coli*: An in-silico DNA marker extraction. Cent. Eur. J. Urol..

[88] Patel S., Mathivanan N., Goyal A. (2017). Bacterial adhesins, the pathogenic weapons to trick host defense arsenal. Biomed. Pharmacother..

[89] Klemm P., Schembri M.A. (2000). Bacterial adhesins: Function and structure. Int. J. Med. Microbiol..

[90] Arciola C.R., Campoccia D., Ravaioli S., Montanaro L. (2015). Polysaccharide intercellular adhesin in biofilm: Structural and regulatory aspects. Front. Cell. Infect. Microbiol..

[91] Bouckaert J., Mackenzie J., De Paz J.L., Chipwaza B., Choudhury D., Zavialov A., Mannerstedt K., Anderson J., Piérard D., Wyns L. (2006). The affinity of the FimH fimbrial adhesin is receptor-driven and quasi-independent of *Escherichia coli* pathotypes. Mol. Microbiol..

[92] Behzadi P., Ranjbar R., Alavian S.M. (2014). Nucleic acid-based approaches for detection of viral hepatitis. Jundishapur J. Microbiol..

[93] Ranjbar R., Behzadi P., Farshad S. (2017). Advances in diagnosis and treatment of Helicobacter pylori infection. Acta Microbiol. Immunol. Hung..

[94] Hospenthal M.K., Costa T.R.D., Waksman G. (2017). A comprehensive guide to pilus biogenesis in Gram-negative bacteria. Nat. Rev. Microbiol..

[95] Ramezanalizadeh F., Owlia P., Rasooli I. (2020). Type I pili, CsuA/B and FimA induce a protective immune response against *Acinetobacter baumannii*. Vaccine.

[96] Behzadi P., Urban E., Matuz M., Benko R., Gajdacs M. (2021). The Role of Gram-Negative Bacteria in Urinary Tract Infections: Current Concepts and Therapeutic Options. Adv. Exp. Med. Biol..

[97] Zhang R., Li D., Fang H., Xie Q., Tang H., Chen L. (2025). Iron-dependent mechanisms in *Acinetobacter baumannii*: Pathogenicity and resistance. JAC-Antimicrob. Resist..

[98] Kim M., Kim D.Y., Song W.Y., Park S.E., Harrison S.A., Chazin W.J., Oh M.H., Kim H.J. (2021). Distinctive roles of two acinetobactin isomers in challenging host nutritional immunity. mBio.

[99] Song W.Y., Kim H.J. (2020). Current biochemical understanding regarding the metabolism of acinetobactin, the major siderophore of the human pathogen *Acinetobacter baumannii*, and outlook for discovery of novel anti-infectious agents based thereon. Nat. Prod. Rep..

[100] Wong D., Nielsen T.B., Bonomo R.A., Pantapalangkoor P., Luna B., Spellberg B. (2017). Clinical and pathophysiological overview of Acinetobacter infections: A century of challenges. Clin. Microbiol. Rev..

[101] Begg S.L. (2019). The role of metal ions in the virulence and viability of bacterial pathogens. Biochem. Soc. Trans..

[102] Obisesan A.O., Zygiel E.M., Nolan E.M. (2021). Bacterial responses to iron withholding by calprotectin. Biochemistry.

[103] Lonergan Z.R., Nairn B.L., Wang J., Hsu Y.-P., Hesse L.E., Beavers W.N., Chazin W.J., Trinidad J.C., VanNieuwenhze M.S., Giedroc D.P. (2019). An *Acinetobacter baumannii*, zinc-regulated peptidase maintains cell wall integrity during immune-mediated nutrient sequestration. Cell Rep..

[104] Hood M.I., Mortensen B.L., Moore J.L., Zhang Y., Kehl-Fie T.E., Sugitani N., Chazin W.J., Caprioli R.M., Skaar E.P. (2012). Identification of an *Acinetobacter baumannii* zinc acquisition system that facilitates resistance to calprotectin-mediated zinc sequestration. PLoS Pathog..

[105] Zschiedrich C.P., Keidel V., Szurmant H. (2016). Molecular Mechanisms of Two-Component Signal Transduction. J. Mol. Biol..

[106] Oh M.H., Islam M.M., Kim N., Choi C.H., Shin M., Shin W.S., Lee J.C. (2025). AbOmpA in *Acinetobacter baumannii*: Exploring virulence mechanisms of outer membrane-integrated and outer membrane vesicle-associated AbOmpA and developing anti-infective agents targeting AbOmpA. J. Biomed. Sci..

[107] Kim N., Son J.H., Kim K., Kim H.J., Kim Y.J., Shin M., Lee J.C. (2021). Global regulator DksA modulates virulence of *Acinetobacter baumannii*. Virulence.

[108] Kim H.-J., Kim N.-Y., Ko S.-Y., Park S.-Y., Oh M.-H., Shin M.-S., Lee Y.-C., Lee J.-C. (2022). Complementary regulation of BfmRS two-component and AbaIR quorum sensing systems to express virulence-associated genes in *Acinetobacter baumannii*. Int. J. Mol. Sci..

[109] Ko S.Y., Kim N., Park S.Y., Kim S.Y., Kim S., Shin M., Lee J.C. (2023). PmrAB controls virulence-associated traits and outer membrane vesicle biogenesis in *Acinetobacter baumannii*. Microb. Pathog..

[110] Kim K., Islam M., Jung H.-w., Lim D., Kim K., Lee S.-G., Park C., Lee J.C., Shin M. (2021). ppGpp signaling plays a critical role in virulence of *Acinetobacter baumannii*. Virulence.

[111] Tacconelli E., Carrara E., Savoldi A., Harbarth S., Mendelson M., Monnet D.L., Pulcini C., Kahlmeter G., Kluytmans J., Carmeli Y. (2018). Discovery, research, and development of new antibiotics: The WHO priority list of antibiotic-resistant bacteria and tuberculosis. Lancet Infect. Dis..

[112] Li P., Zhang S., Wang J., Al-Shamiri M.M., Han B., Chen Y., Han S., Han L. (2023). Uncovering the secretion systems of *Acinetobacter baumannii*: Structures and functions in pathogenicity and antibiotic resistance. Antibiotics.

[113] Hansen F., Porsbo L.J., Frandsen T.H., Kaygisiz A.N.S., Roer L., Henius A.E., Holzknecht B.J., Soes L., Schonning K., Roder B.L. (2023). Characterisation of carbapenemase-producing *Acinetobacter baumannii* isolates from danish patients 2014–2021: Detection of a new international clone—IC11. Int. J. Antimicrob. Agents.

[114] Foudraine D.E., Strepis N., Klaassen C.H.W., Raaphorst M.N., Verbon A., Luider T.M., Goessens W.H.F., Dekker L.J.M. (2021). Rapid and Accurate Detection of Aminoglycoside-Modifying Enzymes and 16S rRNA Methyltransferases by Targeted Liquid Chromatography-Tandem Mass Spectrometry. J. Clin. Microbiol..

[115] de Souza J., D’Espindula H.R.S., Ribeiro I.d.F., Gonçalves G.A., Pillonetto M., Faoro H. (2025). Carbapenem Resistance in *Acinetobacter baumannii*: Mechanisms, Therapeutics, and Innovations. Microorganisms.

[116] Limansky A.S., Mussi M.A., Viale A.M. (2002). Loss of a 29-kilodalton outer membrane protein in *Acinetobacter baumannii* is associated with imipenem resistance. J. Clin. Microbiol..

[117] Han L., Gao Y., Liu Y., Yao S., Zhong S., Zhang S., Wang J., Mi P., Wen Y., Ouyang Z. (2022). An Outer Membrane Protein YiaD Contributes to Adaptive Resistance of Meropenem in *Acinetobacter baumannii*. Microbiol. Spectr..

[118] Kornelsen V., Kumar A. (2021). Update on Multidrug Resistance Efflux Pumps in *Acinetobacter* spp. *Antimicrob*. Agents Chemother..

[119] Martinez-Trejo A., Ruiz-Ruiz J.M., Gonzalez-Avila L.U., Saldana-Padilla A., Hernandez-Cortez C., Loyola-Cruz M.A., Bello-Lopez J.M., Castro-Escarpulli G. (2022). Evasion of Antimicrobial Activity in *Acinetobacter baumannii* by Target Site Modifications: An Effective Resistance Mechanism. Int. J. Mol. Sci..

[120] Bush K., Jacoby G.A., Medeiros A.A. (1995). A functional classification scheme for beta-lactamases and its correlation with molecular structure. Antimicrob. Agents Chemother..

[121] Hall B.G., Barlow M. (2005). Revised Ambler classification of beta-lactamases. J. Antimicrob. Chemother..

[122] Behzadi P., Garcia-Perdomo H.A., Karpinski T.M., Issakhanian L. (2020). Metallo-β-lactamases: A review. Mol. Biol. Rep..

[123] Akinci E., Vahaboglu H. (2010). Minor extended-spectrum beta-lactamases. Expert Rev. Anti-Infect. Ther..

[124] Naas T., Poirel L., Nordmann P. (2008). Minor extended-spectrum beta-lactamases. Clin. Microbiol. Infect. Off. Publ. Eur. Soc. Clin. Microbiol. Infect. Dis..

[125] Behzadi P., Gajdács M., Pallós P., Ónodi B., Stájer A., Matusovits D., Kárpáti K., Burián K., Battah B., Ferrari M. (2022). Relationship between biofilm-formation, phenotypic virulence factors and antibiotic resistance in environmental *Pseudomonas aeruginosa*. Pathogens.

[126] Ferrari C., Corbella M., Gaiarsa S., Comandatore F., Scaltriti E., Bandi C., Cambieri P., Marone P., Sassera D. (2019). Multiple Klebsiella pneumoniae KPC Clones Contribute to an Extended Hospital Outbreak. Front. Microbiol..

[127] Toleman M.A., Simm A.M., Murphy T.A., Gales A.C., Biedenbach D.J., Jones R.N., Walsh T.R. (2002). Molecular characterization of SPM-1, a novel metallo-beta-lactamase isolated in Latin America: Report from the SENTRY antimicrobial surveillance programme. J. Antimicrob. Chemother..

[128] Senda K., Arakawa Y., Nakashima K., Ito H., Ichiyama S., Shimokata K., Kato N., Ohta M. (1996). Multifocal outbreaks of metallo-beta-lactamase-producing *Pseudomonas aeruginosa* resistant to broad-spectrum beta-lactams, including carbapenems. Antimicrob. Agents Chemother..

[129] Poirel L., Naas T., Nicolas D., Collet L., Bellais S., Cavallo J.D., Nordmann P. (2000). Characterization of VIM-2, a carbapenem-hydrolyzing metallo-beta-lactamase and its plasmid- and integron-borne gene from a *Pseudomonas aeruginosa* clinical isolate in France. Antimicrob. Agents Chemother..

[130] Castanheira M., Toleman M.A., Jones R.N., Schmidt F.J., Walsh T.R. (2004). Molecular characterization of a beta-lactamase gene, blaGIM-1, encoding a new subclass of metallo-beta-lactamase. Antimicrob. Agents Chemother..

[131] Lee K., Yum J.H., Yong D., Lee H.M., Kim H.D., Docquier J.D., Rossolini G.M., Chong Y. (2005). Novel acquired metallo-beta-lactamase gene, bla(SIM-1), in a class 1 integron from *Acinetobacter baumannii* clinical isolates from Korea. Antimicrob. Agents Chemother..

[132] Tada T., Shimada K., Satou K., Hirano T., Pokhrel B.M., Sherchand J.B., Kirikae T. (2017). *Pseudomonas aeruginosa* Clinical Isolates in Nepal Coproducing Metallo-beta-Lactamases and 16S rRNA Methyltransferases. Antimicrob. Agents Chemother..

[133] Yong D., Toleman M.A., Bell J., Ritchie B., Pratt R., Ryley H., Walsh T.R. (2012). Genetic and biochemical characterization of an acquired subgroup B3 metallo-beta-lactamase gene, blaAIM-1, and its unique genetic context in *Pseudomonas aeruginosa* from Australia. Antimicrob. Agents Chemother..

[134] El Salabi A., Borra P.S., Toleman M.A., Samuelsen O., Walsh T.R. (2012). Genetic and biochemical characterization of a novel metallo-beta-lactamase, TMB-1, from an Achromobacter xylosoxidans strain isolated in Tripoli, Libya. Antimicrob. Agents Chemother..

[135] Pollini S., Maradei S., Pecile P., Olivo G., Luzzaro F., Docquier J.D., Rossolini G.M. (2013). FIM-1, a new acquired metallo-beta-lactamase from a *Pseudomonas aeruginosa* clinical isolate from Italy. Antimicrob. Agents Chemother..

[136] Jacoby G.A. (2009). AmpC beta-lactamases. Clin. Microbiol. Rev..

[137] Naas T., Oueslati S., Bonnin R.A., Dabos M.L., Zavala A., Dortet L., Retailleau P., Iorga B.I. (2017). Beta-lactamase database (BLDB)–structure and function. J. Enzym. Inhib. Med. Chem..

[138] Poirel L., Naas T., Nordmann P. (2010). Diversity, epidemiology, and genetics of class D beta-lactamases. Antimicrob. Agents Chemother..

[139] Antunes N.T., Lamoureaux T.L., Toth M., Stewart N.K., Frase H., Vakulenko S.B. (2014). Class D beta-lactamases: Are they all carbapenemases?. Antimicrob. Agents Chemother..

[140] Akilli F.M., Ulukanligil M. (2025). Postoperative Meningitis Caused by Multidrug-Resistant Pathogens: A Case Report. New Microbiol..

[141] Ranjbar R., Behzadi P., Mammina C. (2016). Respiratory tularemia: Francisella tularensis and microarray probe designing. Open Microbiol. J..

[142] Migliaccio A., Bray J., Intoccia M., Stabile M., Scala G., Jolley K.A., Brisse S., Zarrilli R. (2023). Phylogenomics of Acinetobacter species and analysis of antimicrobial resistance genes. Front. Microbiol..

[143] Mugnier P.D., Poirel L., Naas T., Nordmann P. (2010). Worldwide dissemination of the blaOXA-23 Carbapenemase gene of *Acinetobacter baumannii*^1^. Emerg. Infect. Dis..

[144] Poirel L., Nordmann P. (2006). Carbapenem resistance in *Acinetobacter baumannii*: Mechanisms and epidemiology. Clin. Microbiol. Infect..

[145] Scaife W., Young H.-K., Paton R.H., Amyes S.G. (1995). Transferable imipenem-resistance in Acinetobacter species from a clinical source. J. Antimicrob. Chemother..

[146] Poirel L., Figueiredo S., Cattoir V., Carattoli A., Nordmann P. (2008). Acinetobacter radioresistens as a silent source of carbapenem resistance for *Acinetobacter* spp. *Antimicrob*. Agents Chemother..

[147] Naas T., Levy M., Hirschauer C., Marchandin H., Nordmann P. (2005). Outbreak of carbapenem-resistant *Acinetobacter baumannii* producing the carbapenemase OXA-23 in a tertiary care hospital of Papeete, French Polynesia. J. Clin. Microbiol..

[148] Corvec S., Poirel L., Naas T., Drugeon H., Nordmann P. (2007). Genetics and expression of the carbapenem-hydrolyzing oxacillinase gene bla OXA-23 in *Acinetobacter baumannii*. Antimicrob. Agents Chemother..

[149] Adams-Haduch J.M., Paterson D.L., Sidjabat H.E., Pasculle A.W., Potoski B.A., Muto C.A., Harrison L.H., Doi Y. (2008). Genetic basis of multidrug resistance in *Acinetobacter baumannii* clinical isolates at a tertiary medical center in Pennsylvania. Antimicrob. Agents Chemother..

[150] Mugnier P.D., Poirel L., Nordmann P. (2009). Functional analysis of insertion sequence IS Aba1, responsible for genomic plasticity of *Acinetobacter baumannii*. J. Bacteriol..

[151] Mugnier P., Poirel L., Pitout M., Nordmann P. (2008). Carbapenem-resistant and OXA-23-producing *Acinetobacter baumannii* isolates in the United Arab Emirates. Clin. Microbiol. Infect..

[152] Mugnier P.D., Bindayna K.M., Poirel L., Nordmann P. (2009). Diversity of plasmid-mediated carbapenem-hydrolysing oxacillinases among carbapenem-resistant *Acinetobacter baumannii* isolates from Kingdom of Bahrain. J. Antimicrob. Chemother..

[153] Bahador A., Raoofian R., Pourakbari B., Taheri M., Hashemizadeh Z., Hashemi F.B. (2015). Genotypic and antimicrobial susceptibility of carbapenem-resistant *Acinetobacter baumannii*: Analysis of is aba elements and bla OXA-23-like genes including a new variant. Front. Microbiol..

[154] Lee C.-R., Lee J.H., Park M., Park K.S., Bae I.K., Kim Y.B., Cha C.-J., Jeong B.C., Lee S.H. (2017). Biology of *Acinetobacter baumannii*: Pathogenesis, antibiotic resistance mechanisms, and prospective treatment options. Front. Cell. Infect. Microbiol..

[155] Fonseca E.L., Scheidegger E., Freitas F.S., Cipriano R., Vicente A.C. (2013). Carbapenem-resistant *Acinetobacter baumannii* from Brazil: Role of carO alleles expression and blaOXA-23 gene. BMC Microbiol..

[156] Gopikrishnan M., George Priya Doss C. (2023). Molecular docking and dynamic approach to screen the drug candidate against the Imipenem-resistant CarO porin in *Acinetobacter baumannii*. Microb. Pathog..

[157] Hwa W.E., Subramaniam G., Mansor M.B., Yan O.S., Gracie, Anbazhagan D., Devi S.S. (2010). Iron regulated outer membrane proteins (IROMPs) as potential targets against carbapenem-resistant *Acinetobacter* spp. isolated from a Medical Centre in Malaysia. Indian J. Med. Res..

[158] Jeong H.W., Cheong H.J., Kim W.J., Kim M.J., Song K.J., Song J.W., Kim H.S., Roh K.H. (2009). Loss of the 29-kilodalton outer membrane protein in the presence of OXA-51-like enzymes in *Acinetobacter baumannii* is associated with decreased imipenem susceptibility. Microb. Drug Resist..

[159] Verma P., Tiwari M., Tiwari V. (2022). Potentiate the activity of current antibiotics by naringin dihydrochalcone targeting the AdeABC efflux pump of multidrug-resistant *Acinetobacter baumannii*. Int. J. Biol. Macromol..

[160] Vahhabi A., Hasani A., Rezaee M.A., Baradaran B., Hasani A., Samadi Kafil H., Abbaszadeh F., Dehghani L. (2021). A plethora of carbapenem resistance in *Acinetobacter baumannii*: No end to a long insidious genetic journey. J. Chemother..

[161] Gehrlein M., Leying H., Cullmann W., Wendt S., Opferkuch W. (1991). Imipenem resistance in Acinetobacter baumanii is due to altered penicillin-binding proteins. Chemotherapy.

[162] Kyriakidis I., Vasileiou E., Pana Z.D., Tragiannidis A. (2021). *Acinetobacter baumannii* Antibiotic Resistance Mechanisms. Pathogens.

[163] Lopez-Hernandez S., Alarcon T., Lopez-Brea M. (1998). Carbapenem resistance mediated by beta-lactamases in clinical isolates of *Acinetobacter baumannii* in Spain. Eur. J. Clin. Microbiol. Infect. Dis..

[164] Afzal-Shah M., Villar H.E., Livermore D.M. (1999). Biochemical characteristics of a carbapenemase from an *Acinetobacter baumannii* isolate collected in Buenos Aires, Argentina. J. Antimicrob. Chemother..

[165] Tsakris A., Tsioni C., Pournaras S., Polyzos S., Maniatis A.N., Sofianou D. (2003). Spread of low-level carbapenem-resistant *Acinetobacter baumannii* clones in a tertiary care Greek hospital. J. Antimicrob. Chemother..

[166] Zarrilli R., Pournaras S., Giannouli M., Tsakris A. (2013). Global evolution of multidrug-resistant *Acinetobacter baumannii* clonal lineages. Int. J. Antimicrob. Agents.

[167] Zander E., Chmielarczyk A., Heczko P., Seifert H., Higgins P.G. (2013). Conversion of OXA-66 into OXA-82 in clinical *Acinetobacter baumannii* isolates and association with altered carbapenem susceptibility. J. Antimicrob. Chemother..

[168] Figueiredo S., Poirel L., Papa A., Koulourida V., Nordmann P. (2009). Overexpression of the naturally occurring blaOXA-51 gene in *Acinetobacter baumannii* mediated by novel insertion sequence ISAba9. Antimicrob. Agents Chemother..

[169] Ahmadi A., Salimizand H. (2017). Delayed identification of *Acinetobacter baumannii* during an outbreak owing to disrupted bla(OXA-51-like) by ISAba19. Int. J. Antimicrob. Agents.

[170] Pournaras S., Markogiannakis A., Ikonomidis A., Kondyli L., Bethimouti K., Maniatis A.N., Legakis N.J., Tsakris A. (2006). Outbreak of multiple clones of imipenem-resistant *Acinetobacter baumannii* isolates expressing OXA-58 carbapenemase in an intensive care unit. J. Antimicrob. Chemother..

[171] Pournaras S., Gogou V., Giannouli M., Dimitroulia E., Dafopoulou K., Tsakris A., Zarrilli R. (2014). Single-locus-sequence-based typing of blaOXA-51-like genes for rapid assignment of *Acinetobacter baumannii* clinical isolates to international clonal lineages. J. Clin. Microbiol..

[172] Aly M., Tayeb H.T., Al Johani S.M., Alyamani E.J., Aldughaishem F., Alabdulkarim I., Balkhy H.H. (2014). Genetic diversity of OXA-51-like genes among multidrug-resistant *Acinetobacter baumannii* in Riyadh, Saudi Arabia. Eur. J. Clin. Microbiol. Infect. Dis..

[173] Quinones Perez D., Aung M.S., Carmona Cartaya Y., Gonzalez Molina M.K., Pereda Novales N., Kobayashi N. (2022). Clonal diversity of Acinetobacter clinical isolates producing NDM-type carbapenemase in Cuba, 2013–2019. IJID Reg..

[174] Bogaerts P., Naas T., Wybo I., Bauraing C., Soetens O., Pierard D., Nordmann P., Glupczynski Y. (2006). Outbreak of infection by carbapenem-resistant *Acinetobacter baumannii* producing the carbapenemase OXA-58 in Belgium. J. Clin. Microbiol..

[175] Vranic-Ladavac M., Bedenic B., Minandri F., Istok M., Bosnjak Z., Francula-Zaninovic S., Ladavac R., Visca P. (2014). Carbapenem resistance and acquired class D beta-lactamases in *Acinetobacter baumannii* from Croatia 2009–2010. Eur. J. Clin. Microbiol. Infect. Dis..

[176] Tsakris A., Ikonomidis A., Pournaras S., Spanakis N., Markogiannakis A. (2006). Carriage of OXA-58 but not of OXA-51 beta-lactamase gene correlates with carbapenem resistance in *Acinetobacter baumannii*. J. Antimicrob. Chemother..

[177] Poirel L., Marque S., Heritier C., Segonds C., Chabanon G., Nordmann P. (2005). OXA-58, a novel class D beta-lactamase involved in resistance to carbapenems in *Acinetobacter baumannii*. Antimicrob. Agents Chemother..

[178] Liakopoulos A., Miriagou V., Katsifas E.A., Karagouni A.D., Daikos G.L., Tzouvelekis L.S., Petinaki E. (2012). Identification of OXA-23-producing *Acinetobacter baumannii* in Greece, 2010 to 2011. Eurosurveillance.

[179] Longjam L.A., Tsering D.C., Das D. (2023). Molecular Characterization of Class A-ESBLs, Class B-MBLs, Class C-AmpC, and Class D-OXA Carbapenemases in MDR *Acinetobacter baumannii* Clinical Isolates in a Tertiary Care Hospital, West Bengal, India. Cureus.

[180] El Bannah A.M.S., Nawar N.N., Hassan R.M.M., Salem S.T.B. (2018). Molecular Epidemiology of Carbapenem-Resistant *Acinetobacter baumannii* in a Tertiary Care Hospital in Egypt: Clonal Spread of blaOXA-23. Microb. Drug Resist..

[181] Dalla-Costa L.M., Coelho J.M., Souza H.A., Castro M.E., Stier C.J., Bragagnolo K.L., Rea-Neto A., Penteado-Filho S.R., Livermore D.M., Woodford N. (2003). Outbreak of carbapenem-resistant *Acinetobacter baumannii* producing the OXA-23 enzyme in Curitiba, Brazil. J. Clin. Microbiol..

[182] Furlan J.P.R., Ramos M.S., Rosa R.D.S., Dos Santos L.D.R., Savazzi E.A., Stehling E.G. (2025). Unveiling transposon-mediated multidrug resistance in OXA-23-producing *Acinetobacter baumannii* ST79/ST233 subclone KL9-OCL10 in Brazil. Gene.

[183] Ababneh Q., Aldaken N., Jaradat Z., Al-Rousan E., Inaya Z., Alsaleh D., Alawneh D., Al Sbei S., Saadoun I. (2025). Predominance of extensively-drug resistant *Acinetobacter baumannii* carrying bla OXA-23 in Jordanian patients admitted to the intensive care units. PLoS ONE.

[184] Pournaras S., Dafopoulou K., Del Franco M., Zarkotou O., Dimitroulia E., Protonotariou E., Poulou A., Zarrilli R., Tsakris A., Greek Study Group on Acinetobacter Antimicrobial R. (2017). Predominance of international clone 2 OXA-23-producing-*Acinetobacter baumannii* clinical isolates in Greece, 2015: Results of a nationwide study. Int. J. Antimicrob. Agents.

[185] Karampatakis T., Tsergouli K., Politi L., Diamantopoulou G., Iosifidis E., Antachopoulos C., Karyoti A., Sdougka M., Tsakris A., Roilides E. (2019). Polyclonal predominance of concurrently producing OXA-23 and OXA-58 carbapenem-resistant *Acinetobacter baumannii* strains in a pediatric intensive care unit. Mol. Biol. Rep..

[186] Liu H., Liu X., He J., Zhang L., Zhao F., Zhou Z., Hua X., Yu Y. (2023). Emergence and Evolution of OXA-23-Producing ST46(Pas)-ST462(Oxf)-KL28-OCL1 Carbapenem-Resistant *Acinetobacter baumannii* Mediated by a Novel ISAba1-Based Tn7534 Transposon. Antibiotics.

[187] Carascal M.B., Destura R.V., Rivera W.L. (2024). Colorimetric Loop-Mediated Isothermal Amplification Assays Accurately Detect bla(OXA-23-like) and ISAba1 Genes from *Acinetobacter baumannii* in Pure Cultures and Spiked Human Sera. Microb. Drug Resist..

[188] Aranzamendi M., Xanthopoulou K., Sanchez-Urtaza S., Burgwinkel T., Arazo Del Pino R., Lucassen K., Perez-Vazquez M., Oteo-Iglesias J., Sota M., Marimon J.M. (2024). Genomic Surveillance Uncovers a 10-Year Persistence of an OXA-24/40 *Acinetobacter baumannii* Clone in a Tertiary Hospital in Northern Spain. Int. J. Mol. Sci..

[189] Grosso F., Quinteira S., Peixe L. (2011). Understanding the dynamics of imipenem-resistant *Acinetobacter baumannii* lineages within Portugal. Clin. Microbiol. Infect. Off. Publ. Eur. Soc. Clin. Microbiol. Infect. Dis..

[190] Li J., Fu Y., Zhang J., Zhao Y., Fan X., Yu L., Wang Y., Zhang X., Li C. (2020). The efficacy of colistin monotherapy versus combination therapy with other antimicrobials against carbapenem-resistant *Acinetobacter baumannii* ST2 isolates. J. Chemother..

[191] Iovleva A., Mustapha M.M., Griffith M.P., Komarow L., Luterbach C., Evans D.R., Cober E., Richter S.S., Rydell K., Arias C.A. (2022). Carbapenem-resistant *Acinetobacter baumannii* in US hospitals: Diversification of circulating lineages and antimicrobial resistance. MBio.

[192] Mavroidi A., Likousi S., Palla E., Katsiari M., Roussou Z., Maguina A., Platsouka E.D. (2015). Molecular identification of tigecycline-and colistin-resistant carbapenemase-producing *Acinetobacter baumannii* from a Greek hospital from 2011 to 2013. J. Med. Microbiol..

[193] Tsakris A., Ikonomidis A., Pournaras S., Tzouvelekis L.S., Sofianou D., Legakis N.J., Maniatis A.N. (2006). VIM-1 metallo-beta-lactamase in *Acinetobacter baumannii*. Emerg. Infect. Dis..

[194] Loli A., Tzouvelekis L.S., Gianneli D., Tzelepi E., Miriagou V. (2008). Outbreak of *Acinetobacter baumannii* with chromosomally encoded VIM-1 undetectable by imipenem-EDTA synergy tests. Antimicrob. Agents Chemother..

[195] Tsakris A., Ikonomidis A., Poulou A., Spanakis N., Vrizas D., Diomidous M., Pournaras S., Markou F. (2008). Clusters of imipenem-resistant *Acinetobacter baumannii* clones producing different carbapenemases in an intensive care unit. Clin. Microbiol. Infect. Off. Publ. Eur. Soc. Clin. Microbiol. Infect. Dis..

[196] Peleg A.Y., Bell J.M., Hofmeyr A., Wiese P. (2006). Inter-country transfer of Gram-negative organisms carrying the VIM-4 and OXA-58 carbapenem-hydrolysing enzymes. J. Antimicrob. Chemother..

[197] Yum J.H., Yi K., Lee H., Yong D., Lee K., Kim J.M., Rossolini G.M., Chong Y. (2002). Molecular characterization of metallo-beta-lactamase-producing *Acinetobacter baumannii* and Acinetobacter genomospecies 3 from Korea: Identification of two new integrons carrying the bla(VIM-2) gene cassettes. J. Antimicrob. Chemother..

[198] Oh E.J., Lee S., Park Y.J., Park J.J., Park K., Kim S.I., Kang M.W., Kim B.K. (2003). Prevalence of metallo-beta-lactamase among *Pseudomonas aeruginosa* and *Acinetobacter baumannii* in a Korean university hospital and comparison of screening methods for detecting metallo-beta-lactamase. J. Microbiol. Methods.

[199] Castanheira M., Costello S.E., Woosley L.N., Deshpande L.M., Davies T.A., Jones R.N. (2014). Evaluation of clonality and carbapenem resistance mechanisms among *Acinetobacter baumannii*-Acinetobacter calcoaceticus complex and Enterobacteriaceae isolates collected in European and Mediterranean countries and detection of two novel beta-lactamases, GES-22 and VIM-35. Antimicrob. Agents Chemother..

[200] Sung J.Y., Kwon K.C., Park J.W., Kim Y.S., Kim J.M., Shin K.S., Kim J.W., Ko C.S., Shin S.Y., Song J.H. (2008). Dissemination of IMP-1 and OXA type beta-lactamase in carbapenem-resistant *Acinetobacter baumannii*. Korean J. Lab. Med..

[201] Kim H.J., Kim H.S., Lee J.M., Yoon S.S., Yong D. (2016). Rapid detection of *Pseudomonas aeruginosa* and *Acinetobacter baumannii* Harboring bla(VIM-2), bla(IMP-1) and bla(OXA-23) genes by using loop-mediated isothermal amplification methods. Ann. Lab. Med..

[202] Davoodi S., Boroumand M.A., Sepehriseresht S., Pourgholi L. (2015). Detection of VIM- and IMP-type Metallo-Beta-Lactamase Genes in *Acinetobacter baumannii* Isolates from Patients in Two Hospitals in Tehran. Iran. J. Biotechnol..

[203] Massik A., Hibaoui L., Benboubker M., Yahyaoui G., Oumokhtar B., Mahmoud M. (2023). *Acinetobacter baumannii* Carbapenemase Producers in Morocco: Genetic Diversity. Cureus.

[204] Massik A., Hibaoui L., Moussa B., Yahyaoui G., Oumokhtar B., Mahmoud M. (2022). First report of SPM metallo-beta-lactamases producing *Acinetobacter baumannii* isolates in Morocco. Iran. J. Microbiol..

[205] Ferjani S., Kanzari L., Maamar E., Hamzaoui Z., Rehaiem A., Ferjani A., Boutiba-Ben Boubaker I. (2022). Extensively drug-resistant *Acinetobacter baumannii* co-producing VIM-2 and OXA-23 in intensive care units: Results of a one-day point prevalence in a Tunisian hospital. Infect. Dis. Now..

[206] Zhou Z., Guan R., Yang Y., Chen L., Fu J., Deng Q., Xie Y., Huang Y., Wang J., Wang D. (2012). Identification of New Delhi metallo-beta-lactamase gene (NDM-1) from a clinical isolate of Acinetobacter junii in China. Can. J. Microbiol..

[207] Ghazawi A., Sonnevend A., Bonnin R.A., Poirel L., Nordmann P., Hashmey R., Rizvi T.A., M B.H., Pal T. (2012). NDM-2 carbapenemase-producing *Acinetobacter baumannii* in the United Arab Emirates. Clin. Microbiol. Infect. Off. Publ. Eur. Soc. Clin. Microbiol. Infect. Dis..

[208] Revathi G., Siu L.K., Lu P.L., Huang L.Y. (2013). First report of NDM-1-producing *Acinetobacter baumannii* in East Africa. Int. J. Infect. Dis..

[209] Decousser J.W., Jansen C., Nordmann P., Emirian A., Bonnin R.A., Anais L., Merle J.C., Poirel L. (2013). Outbreak of NDM-1-producing *Acinetobacter baumannii* in France, January to May 2013. Eurosurveillance.

[210] Travi G., Peracchi F., Merli M., Lo Re N., Matarazzo E., Tartaglione L., Bielli A., Casalicchio G., Crippa F., Vismara C.S. (2024). Cefiderocol-Based Regimen for Acinetobacter NDM-1 Outbreak. Antibiotics.

[211] Voulgari E., Politi L., Pitiriga V., Dendrinos J., Poulou A., Georgiadis G., Tsakris A. (2016). First report of an NDM-1 metallo-beta-lactamase-producing *Acinetobacter baumannii* clinical isolate in Greece. Int. J. Antimicrob. Agents.

[212] Chatterjee S., Datta S., Roy S., Ramanan L., Saha A., Viswanathan R., Som T., Basu S. (2016). Carbapenem Resistance in *Acinetobacter baumannii* and Other *Acinetobacter* spp. Causing Neonatal Sepsis: Focus on NDM-1 and Its Linkage to ISAba125. Front. Microbiol..

[213] Xanthopoulou K., Urrutikoetxea-Gutierrez M., Vidal-Garcia M., Diaz de Tuesta Del Arco J.L., Sanchez-Urtaza S., Wille J., Seifert H., Higgins P.G., Gallego L. (2020). First Report of New Delhi Metallo-beta-Lactamase-6 (NDM-6) in a Clinical *Acinetobacter baumannii* Isolate From Northern Spain. Front. Microbiol..

[214] Gaillot S., Oueslati S., Vuillemenot J.B., Bour M., Iorga B.I., Triponney P., Plesiat P., Bonnin R.A., Naas T., Jeannot K. (2023). Genomic characterization of an NDM-9-producing *Acinetobacter baumannii* clinical isolate and role of Glu152Lys substitution in the enhanced cefiderocol hydrolysis of NDM-9. Front. Microbiol..

[215] Kitti T., Manrueang S., Leungtongkam U., Khongfak S., Thummeepak R., Wannalerdsakun S., Jindayok T., Sitthisak S. (2023). Genomic relatedness and dissemination of bla (NDM-5) among *Acinetobacter baumannii* isolated from hospital environments and clinical specimens in Thailand. PeerJ.

[216] Gutierrez K., Vasquez-Mendoza A., Rodriguez C. (2024). An outbreak of severe or lethal infections by a multidrug-resistant *Acinetobacter baumannii* ST126 strain carrying a plasmid with bla(NDM-1) and bla(OXA-58) carbapenemases. Diagn. Microbiol. Infect. Dis..

[217] Joshi P.R., Acharya M., Kakshapati T., Leungtongkam U., Thummeepak R., Sitthisak S. (2017). Co-existence of bla(OXA-23) and bla(NDM-1) genes of *Acinetobacter baumannii* isolated from Nepal: Antimicrobial resistance and clinical significance. Antimicrob. Resist. Infect. Control.

[218] Lukovic B., Gajic I., Dimkic I., Kekic D., Zornic S., Pozder T., Radisavljevic S., Opavski N., Kojic M., Ranin L. (2020). The first nationwide multicenter study of *Acinetobacter baumannii* recovered in Serbia: Emergence of OXA-72, OXA-23 and NDM-1-producing isolates. Antimicrob. Resist. Infect. Control.

[219] Martins-Goncalves T., Pimenta J.S., Fontana H., Esposito F., Melocco G., Dantas K., Vasquez-Ponce F., Carrara F.E., Vespero E.C., Lincopan N. (2024). *Acinetobacter baumannii* international clone 2 co-producing OXA-23, NDM-1, and ArmA emerging in South America. Antimicrob. Agents Chemother..

[220] Hidalgo E., Sotelo J., Perez-Vazquez M., Iniesta A., Canada-Garcia J.E., Valiente O., Aracil B., Arana D.M., Oteo-Iglesias J. (2025). Emergence of NDM-1- and OXA-23-Co-Producing *Acinetobacter baumannii* ST1 Isolates from a Burn Unit in Spain. Microorganisms.

[221] Castilho P.O.S., Takahashi F.M., Onca Moreira M.J., Martins-Goncalves T., Carrara F.E., Lincopan N., Vespero E.C. (2025). Outbreak and persistence of dual carbapenemase (OXA-23 and NDM-1)-producing *Acinetobacter baumannii* international clone 2 (ST2) in a tertiary hospital intensive care unit in Brazil. J. Hosp. Infect..

[222] Robledo I.E., Aquino E.E., Sante M.I., Santana J.L., Otero D.M., Leon C.F., Vazquez G.J. (2010). Detection of KPC in *Acinetobacter* spp. in Puerto Rico. Antimicrob. Agents Chemother..

[223] Escandon-Vargas K., Reyes S., Gutierrez S., Villegas M.V. (2017). The epidemiology of carbapenemases in Latin America and the Caribbean. Expert Rev. Anti-Infect. Ther..

[224] Caneiras C., Calisto F., Jorge da Silva G., Lito L., Melo-Cristino J., Duarte A. (2018). First Description of Colistin and Tigecycline-Resistant *Acinetobacter baumannii* Producing KPC-3 Carbapenemase in Portugal. Antibiotics.

[225] Miniaoui D., Dziri O., Ben Lamine Y., El Salabi A.A., Omar E.O., Slimene K., Dziri R., Bouhalila-Besbes S., Hadjadj L., Mabrouk A. (2023). Prevalence of carbapenemases among Gram-negative bacteria in Tunisia: First report of KPC-2 producing *Acinetobacter baumannii*. J. Infect. Dev. Ctries..

[226] Gaiarsa S., Batisti Biffignandi G., Esposito E.P., Castelli M., Jolley K.A., Brisse S., Sassera D., Zarrilli R. (2019). Comparative analysis of the two *Acinetobacter baumannii* multilocus sequence typing (MLST) schemes. Front. Microbiol..

[227] Bialek-Davenet S., Criscuolo A., Ailloud F., Passet V., Jones L., Delannoy-Vieillard A.-S., Garin B., Le Hello S., Arlet G., Nicolas-Chanoine M.-H. (2014). Genomic definition of hypervirulent and multidrug-resistant Klebsiella pneumoniae clonal groups. Emerg. Infect. Dis..

[228] Bartual S.G., Seifert H., Hippler C., Luzon M.A.D.n., Wisplinghoff H., Rodríguez-Valera F. (2005). Development of a multilocus sequence typing scheme for characterization of clinical isolates of *Acinetobacter baumannii*. J. Clin. Microbiol..

[229] Wisplinghoff H., Hippler C., Bartual S., Haefs C., Stefanik D., Higgins P., Seifert H. (2008). Molecular epidemiology of clinical *Acinetobacter baumannii* and Acinetobacter genomic species 13TU isolates using a multilocus sequencing typing scheme. Clin. Microbiol. Infect..

[230] Diancourt L., Passet V., Nemec A., Dijkshoorn L., Brisse S. (2010). The population structure of *Acinetobacter baumannii*: Expanding multiresistant clones from an ancestral susceptible genetic pool. PLoS ONE.

[231] Da Silva G., Van Der Reijden T., Domingues S., Mendonça N., Petersen K., Dijkshoorn L. (2014). Characterization of a novel international clonal complex (CC32) of *Acinetobacter baumannii* with epidemic potential. Epidemiol. Infect..

[232] Sahl J.W., Del Franco M., Pournaras S., Colman R.E., Karah N., Dijkshoorn L., Zarrilli R. (2015). Phylogenetic and genomic diversity in isolates from the globally distributed *Acinetobacter baumannii* ST25 lineage. Sci. Rep..

[233] Ou H.-Y., Kuang S.N., He X., Molgora B.M., Ewing P.J., Deng Z., Osby M., Chen W., Xu H.H. (2015). Complete genome sequence of hypervirulent and outbreak-associated *Acinetobacter baumannii* strain LAC-4: Epidemiology, resistance genetic determinants and potential virulence factors. Sci. Rep..

[234] Feng Y., Ruan Z., Shu J., Chen C.-L., Chiu C.-H. (2016). A glimpse into evolution and dissemination of multidrug-resistant *Acinetobacter baumannii* isolates in East Asia: A comparative genomics study. Sci. Rep..

[235] Tomaschek F., Higgins P.G., Stefanik D., Wisplinghoff H., Seifert H. (2016). Head-to-head comparison of two multi-locus sequence typing (MLST) schemes for characterization of *Acinetobacter baumannii* outbreak and sporadic isolates. PLoS ONE.

[236] Hamidian M., Nigro S.J., Hall R.M. (2017). Problems with the Oxford multilocus sequence typing scheme for *Acinetobacter baumannii*: Do sequence type 92 (ST92) and ST109 exist?. J. Clin. Microbiol..

[237] Hamouda A., Evans B.A., Towner K.J., Amyes S.G. (2010). Characterization of epidemiologically unrelated *Acinetobacter baumannii* isolates from four continents by use of multilocus sequence typing, pulsed-field gel electrophoresis, and sequence-based typing of bla OXA-51-like genes. J. Clin. Microbiol..

[238] Jolley K.A., Bray J.E., Maiden M.C. (2018). Open-access bacterial population genomics: BIGSdb software, the PubMLST. org website and their applications. Wellcome Open Res..

[239] Gogou V., Pournaras S., Giannouli M., Voulgari E., Piperaki E.T., Zarrilli R., Tsakris A. (2011). Evolution of multidrug-resistant *Acinetobacter baumannii* clonal lineages: A 10 year study in Greece (2000-09). J. Antimicrob. Chemother..

[240] Li S., Jiang G., Wang S., Wang M., Wu Y., Zhang J., Liu X., Zhong L., Zhou M., Xie S. (2025). Emergence and global spread of a dominant multidrug-resistant clade within *Acinetobacter baumannii*. Nat. Commun..

[241] Rodgers D., Pasteran F., Calderon M., Jaber S., Traglia G.M., Albornoz E., Corso A., Vila A.J., Bonomo R.A., Adams M.D. (2020). Characterisation of ST25 NDM-1-producing *Acinetobacter* spp. strains leading the increase in NDM-1 emergence in Argentina. J. Glob. Antimicrob. Resist..

[242] Aung M.S., Hlaing M.S., San N., Aung M.T., Mar T.T., Kobayashi N. (2021). Clonal diversity of *Acinetobacter baumannii* clinical isolates in Myanmar: Identification of novel ST1407 harbouring blaNDM-1. New Microbes New Infect..

[243] Wang K., Zhu W., Gong L., Yang X., Ye H., Lou Z., Yang J., Jiang X., Li W., Tao F. (2025). Genomic and phenotypic insights into ST164 bla(NDM-1)-positive *Acinetobacter baumannii* from intestinal colonization in China. BMC Microbiol..

[244] Heydari F., Mammina C., Koksal F. (2015). NDM-1-producing *Acinetobacter baumannii* ST85 now in Turkey, including one isolate from a Syrian refugee. J. Med. Microbiol..

[245] Bonnin R.A., Cuzon G., Poirel L., Nordmann P. (2013). Multidrug-resistant *Acinetobacter baumannii* clone, France. Emerg. Infect. Dis..

[246] Luo T.L., Martin M.J., Kovalchuk V., Kondratiuk V., Trapaidze N., Metreveli M., Hulseberg C.E., Dao H.D., Kwak Y.I., Maybank R. (2024). Detection of carbapenemase producing *Acinetobacter baumannii* ST19 from Georgia and Ukraine carrying bla (OXA-23), bla (OXA-72), and/or bla (NDM-5), December 2019 to June 2023. Eurosurveillance.

[247] Nawfal Dagher T., Hadjadj L., Bittar F., Fenianos F., Abdo E., Rolain J.M., Al-Bayssari C. (2025). Clonal dissemination of an NDM-2-producing *Acinetobacter baumannii* ST103 clone resulting in an outbreak in an intensive care unit of a Lebanese tertiary care hospital. J. Glob. Antimicrob. Resist..

[248] Yusuf E., Bax H.I., Verkaik N.J., van Westreenen M. (2021). An Update on Eight “New” Antibiotics against Multidrug-Resistant Gram-Negative Bacteria. J. Clin. Med..

[249] Paul M., Carrara E., Retamar P., Tangden T., Bitterman R., Bonomo R.A., de Waele J., Daikos G.L., Akova M., Harbarth S. (2022). European Society of Clinical Microbiology and Infectious Diseases (ESCMID) guidelines for the treatment of infections caused by multidrug-resistant Gram-negative bacilli (endorsed by European society of intensive care medicine). Clin. Microbiol. Infect. Off. Publ. Eur. Soc. Clin. Microbiol. Infect. Dis..

[250] Biswas S., Brunel J.M., Dubus J.C., Reynaud-Gaubert M., Rolain J.M. (2012). Colistin: An update on the antibiotic of the 21st century. Expert Rev. Anti-Infect. Ther..

[251] PubChem Compound Summary for CID 44144393. Colistin. https://pubchem.ncbi.nlm.nih.gov/compound/Colistine.

[252] Knox C., Wilson M., Klinger C.M., Franklin M., Oler E., Wilson A., Pon A., Cox J., Chin N.E., Strawbridge S.A. (2024). DrugBank 6.0: The DrugBank knowledgebase for 2024. Nucleic Acids Res..

[253] PubChem Compound Summary for CID 18541918. Ampicillin-Sulbactam. https://pubchem.ncbi.nlm.nih.gov/compound/Ampicillin-sulbactam.

[254] PubChem Compound Summary for CID 54686904. Tigecycline. https://pubchem.ncbi.nlm.nih.gov/compound/Tigecycline.

[255] PubChem Compound Summary for CID 446987. Fosfomycin. https://pubchem.ncbi.nlm.nih.gov/compound/Fosfomycin.

[256] PubChem Compound Summary for CID 42613186. Plazomicin. https://pubchem.ncbi.nlm.nih.gov/compound/Plazomicin.

[257] PubChem Compound Summary for CID 54726192. Eravacycline. https://pubchem.ncbi.nlm.nih.gov/compound/Tp-434.

[258] PubChem Compound Summary for CID 77843966. Cefiderocol. https://pubchem.ncbi.nlm.nih.gov/compound/Cefiderocol.

[259] PubChem Compound Summary for CID 171758. Temocillin. https://pubchem.ncbi.nlm.nih.gov/compound/Temocillin.

[260] PubChem Compound Summary for CID 172973390. Ceftolozane-Tazobactam. https://pubchem.ncbi.nlm.nih.gov/compound/Ceftolozane-tazobactam.

[261] PubChem Compound Summary for CID 86298703. Meropenem; Vaborbactam. https://pubchem.ncbi.nlm.nih.gov/compound/86298703.

[262] PubChem Compound Summary for CID 90643431. Avycaz. https://pubchem.ncbi.nlm.nih.gov/compound/Avycaz.

[263] Cassir N., Rolain J.M., Brouqui P. (2014). A new strategy to fight antimicrobial resistance: The revival of old antibiotics. Front. Microbiol..

[264] Akajagbor D.S., Wilson S.L., Shere-Wolfe K.D., Dakum P., Charurat M.E., Gilliam B.L. (2013). Higher incidence of acute kidney injury with intravenous colistimethate sodium compared with polymyxin B in critically ill patients at a tertiary care medical center. Clin. Infect. Dis..

[265] Vardakas K.Z., Athanassaki F., Pitiriga V., Falagas M.E. (2019). Clinical relevance of in vitro synergistic activity of antibiotics for multidrug-resistant Gram-negative infections: A systematic review. J. Glob. Antimicrob. Resist..

[266] Paul M., Daikos G.L., Durante-Mangoni E., Yahav D., Carmeli Y., Benattar Y.D., Skiada A., Andini R., Eliakim-Raz N., Nutman A. (2018). Colistin alone versus colistin plus meropenem for treatment of severe infections caused by carbapenem-resistant Gram-negative bacteria: An open-label, randomised controlled trial. Lancet Infect. Dis..

[267] Durante-Mangoni E., Signoriello G., Andini R., Mattei A., De Cristoforo M., Murino P., Bassetti M., Malacarne P., Petrosillo N., Galdieri N. (2013). Colistin and rifampicin compared with colistin alone for the treatment of serious infections due to extensively drug-resistant *Acinetobacter baumannii*: A multicenter, randomized clinical trial. Clin. Infect. Dis..

[268] Dickstein Y., Lellouche J., Ben Dalak Amar M., Schwartz D., Nutman A., Daitch V., Yahav D., Leibovici L., Skiada A., Antoniadou A. (2019). Treatment outcomes of colistin-and carbapenem-resistant *Acinetobacter baumannii* infections: An exploratory subgroup analysis of a randomized clinical trial. Clin. Infect. Dis..

[269] Betrosian A.P., Frantzeskaki F., Xanthaki A., Douzinas E.E. (2008). Efficacy and safety of high-dose ampicillin/sulbactam vs. colistin as monotherapy for the treatment of multidrug resistant *Acinetobacter baumannii* ventilator-associated pneumonia. J. Infect..

[270] Zalts R., Neuberger A., Hussein K., Raz-Pasteur A., Geffen Y., Mashiach T., Finkelstein R. (2016). Treatment of Carbapenem-Resistant *Acinetobacter baumannii* Ventilator-Associated Pneumonia: Retrospective Comparison Between Intravenous Colistin and Intravenous Ampicillin-Sulbactam. Am. J. Ther..

[271] Mosaed R., Haghighi M., Kouchak M., Miri M.M., Salarian S., Shojaei S., Javadi A., Taheri S., Nazirzadeh P., Foroumand M. (2018). Interim Study: Comparison Of Safety And Efficacy of Levofloxacin Plus Colistin Regimen With Levofloxacin Plus High Dose Ampicillin/Sulbactam Infusion In Treatment of Ventilator-Associated Pneumonia Due To Multi Drug Resistant Acinetobacter. Iran. J. Pharm. Res..

[272] Safarika A., Galani I., Pistiki A., Giamarellos-Bourboulis E.J. (2015). Time-kill effect of levofloxacin on multidrug-resistant *Pseudomonas aeruginosa* and *Acinetobacter baumannii*: Synergism with imipenem and colistin. Eur. J. Clin. Microbiol. Infect. Dis..

[273] Choi S.J., Kim E.S. (2024). Optimizing Treatment for Carbapenem-Resistant *Acinetobacter baumannii* Complex Infections: A Review of Current Evidence. Infect. Chemother..

[274] Lawandi A., Yek C., Kadri S.S. (2022). IDSA guidance and ESCMID guidelines: Complementary approaches toward a care standard for MDR Gram-negative infections. Clin. Microbiol. Infect. Off. Publ. Eur. Soc. Clin. Microbiol. Infect. Dis..

[275] Kubin C.J., Garzia C., Uhlemann A.-C. (2025). *Acinetobacter baumannii* treatment strategies: A review of therapeutic challenges and considerations. Antimicrob. Agents Chemother..

[276] Shields R.K., Paterson D.L., Tamma P.D. (2023). Navigating available treatment options for carbapenem-resistant *Acinetobacter baumannii*-calcoaceticus complex infections. Clin. Infect. Dis..

[277] Karruli A., Migliaccio A., Pournaras S., Durante-Mangoni E., Zarrilli R. (2023). Cefiderocol and sulbactam-durlobactam against carbapenem-resistant *Acinetobacter baumannii*. Antibiotics.

[278] Keam S.J. (2023). Sulbactam/durlobactam: First approval. Drugs.

[279] Kaye K.S., Shorr A.F., Wunderink R.G., Du B., Poirier G.E., Rana K., Miller A., Lewis D., O’Donnell J., Chen L. (2023). Efficacy and safety of sulbactam–durlobactam versus colistin for the treatment of patients with serious infections caused by *Acinetobacter baumannii*–calcoaceticus complex: A multicentre, randomised, active-controlled, phase 3, non-inferiority clinical trial (ATTACK). Lancet Infect. Dis..

[280] Seifert H., Müller C., Stefanik D., Higgins P.G., Miller A., Kresken M. (2020). In vitro activity of sulbactam/durlobactam against global isolates of carbapenem-resistant *Acinetobacter baumannii*. J. Antimicrob. Chemother..

[281] Entenza J.M., Moreillon P. (2009). Tigecycline in combination with other antimicrobials: A review of in vitro, animal and case report studies. Int. J. Antimicrob. Agents.

[282] Karageorgopoulos D.E., Kelesidis T., Kelesidis I., Falagas M.E. (2008). Tigecycline for the treatment of multidrug-resistant (including carbapenem-resistant) Acinetobacter infections: A review of the scientific evidence. J. Antimicrob. Chemother..

[283] Chuang Y.C., Cheng C.Y., Sheng W.H., Sun H.Y., Wang J.T., Chen Y.C., Chang S.C. (2014). Effectiveness of tigecycline-based versus colistin- based therapy for treatment of pneumonia caused by multidrug-resistant *Acinetobacter baumannii* in a critical setting: A matched cohort analysis. BMC Infect. Dis..

[284] Kim W.Y., Moon J.Y., Huh J.W., Choi S.H., Lim C.M., Koh Y., Chong Y.P., Hong S.B. (2016). Comparable Efficacy of Tigecycline versus Colistin Therapy for Multidrug-Resistant and Extensively Drug-Resistant *Acinetobacter baumannii* Pneumonia in Critically Ill Patients. PLoS ONE.

[285] Kwon S.H., Ahn H.L., Han O.Y., La H.O. (2014). Efficacy and safety profile comparison of colistin and tigecycline on the extensively drug resistant *Acinetobacter baumannii*. Biol. Pharm. Bull..

[286] Ku K., Pogue J.M., Moshos J., Bheemreddy S., Wang Y., Bhargava A., Campbell M., Khandker N., Lephart P.R., Chopra T. (2012). Retrospective evaluation of colistin versus tigecycline for the treatment of *Acinetobacter baumannii* and/or carbapenem-resistant Enterobacteriaceae infections. Am. J. Infect. Control.

[287] Ye J.J., Lin H.S., Yeh C.F., Wu Y.M., Huang P.Y., Yang C.C., Huang C.T., Lee M.H. (2016). Tigecycline-based versus sulbactam-based treatment for pneumonia involving multidrug-resistant Acinetobacter calcoaceticus-*Acinetobacter baumannii* complex. BMC Infect. Dis..

[288] Niu T., Luo Q., Li Y., Zhou Y., Yu W., Xiao Y. (2019). Comparison of Tigecycline or Cefoperazone/Sulbactam therapy for bloodstream infection due to Carbapenem-resistant *Acinetobacter baumannii*. Antimicrob. Resist. Infect. Control.

[289] Mezzatesta M.L., La Rosa G., Maugeri G., Zingali T., Caio C., Novelli A., Stefani S. (2017). In vitro activity of fosfomycin trometamol and other oral antibiotics against multidrug-resistant uropathogens. Int. J. Antimicrob. Agents.

[290] Behzadi P., Garcia-Perdomo H.A., Autran Gomez A.M., Pinheiro M., Sarshar M. (2023). Editorial: Uropathogens, urinary tract infections, the host-pathogen interactions and treatment. Front. Microbiol..

[291] Issakhanian L., Behzadi P. (2019). Antimicrobial Agents and Urinary Tract Infections. Curr. Pharm. Des..

[292] Assimakopoulos S.F., Karamouzos V., Eleftheriotis G., Lagadinou M., Bartzavali C., Kolonitsiou F., Paliogianni F., Fligou F., Marangos M. (2023). Efficacy of Fosfomycin-Containing Regimens for Treatment of Bacteremia Due to Pan-Drug Resistant *Acinetobacter baumannii* in Critically Ill Patients: A Case Series Study. Pathogens.

[293] Russo A., Gulli S.P., D’Avino A., Borrazzo C., Carannante N., Dezza F.C., Covino S., Polistina G., Fiorentino G., Trecarichi E.M. (2024). Intravenous fosfomycin for treatment of severe infections caused by carbapenem-resistant *Acinetobacter baumannii*: A multi-centre clinical experience. Int. J. Antimicrob. Agents.

[294] Garcia-Salguero C., Rodriguez-Avial I., Picazo J.J., Culebras E. (2015). Can Plazomicin Alone or in Combination Be a Therapeutic Option against Carbapenem-Resistant *Acinetobacter baumannii*?. Antimicrob. Agents Chemother..

[295] Eljaaly K., Alharbi A., Alshehri S., Ortwine J.K., Pogue J.M. (2019). Plazomicin: A Novel Aminoglycoside for the Treatment of Resistant Gram-Negative Bacterial Infections. Drugs.

[296] Jackson M.N.W., Wei W., Mang N.S., Prokesch B.C., Ortwine J.K. (2024). Combination eravacycline therapy for ventilator-associated pneumonia due to carbapenem-resistant *Acinetobacter baumannii* in patients with COVID-19: A case series. Pharmacotherapy.

[297] Zhanel G.G., Golden A.R., Zelenitsky S., Wiebe K., Lawrence C.K., Adam H.J., Idowu T., Domalaon R., Schweizer F., Zhanel M.A. (2019). Cefiderocol: A Siderophore Cephalosporin with Activity Against Carbapenem-Resistant and Multidrug-Resistant Gram-Negative Bacilli. Drugs.

[298] Shortridge D., Streit J.M., Mendes R., Castanheira M. (2022). In Vitro Activity of Cefiderocol against U.S. and European Gram-Negative Clinical Isolates Collected in 2020 as Part of the SENTRY Antimicrobial Surveillance Program. Microbiol. Spectr..

[299] Soriano A., Mensa J. (2022). Mechanism of action of cefiderocol. Rev. Esp. Quim..

[300] Falcone M., Tiseo G., Nicastro M., Leonildi A., Vecchione A., Casella C., Forfori F., Malacarne P., Guarracino F., Barnini S. (2021). Cefiderocol as Rescue Therapy for *Acinetobacter baumannii* and Other Carbapenem-resistant Gram-negative Infections in Intensive Care Unit Patients. Clin. Infect. Dis..

[301] Bassetti M., Echols R., Matsunaga Y., Ariyasu M., Doi Y., Ferrer R., Lodise T.P., Naas T., Niki Y., Paterson D.L. (2021). Efficacy and safety of cefiderocol or best available therapy for the treatment of serious infections caused by carbapenem-resistant Gram-negative bacteria (CREDIBLE-CR): A randomised, open-label, multicentre, pathogen-focused, descriptive, phase 3 trial. Lancet Infect. Dis..

[302] Desmoulin A., Sababadichetty L., Kamus L., Daniel M., Feletti L., Allou N., Potron A., Leroy A.G., Jaffar-Bandjee M.C., Belmonte O. (2024). Adaptive resistance to cefiderocol in carbapenem-resistant *Acinetobacter baumannii* (CRAB): Microbiological and clinical issues. Heliyon.

[303] Livermore D.M., Tulkens P.M. (2009). Temocillin revived. J. Antimicrob. Chemother..

[304] Shafiekhani M., Fatemi S.A., Hosseini P., Marhemati F., Mohammadi S., Sharifi F., Moorkani Kurde Esfahani Pour A., Sadeghi Habibabad F., Saad Abadi N., Shorafa E. (2023). Pharmacokinetic and Pharmacodynamic Considerations of Novel Antibiotic Agents for Pediatric Infections: A Narrative Review. Surg. Infect..

[305] Jun S.H., Lee D.E., Hwang H.R., Kim N., Kim H.J., Lee Y.C., Kim Y.K., Lee J.C. (2021). Clonal change of carbapenem-resistant *Acinetobacter baumannii* isolates in a Korean hospital. Infect. Genet. Evol..

[306] Ju Y.G., Lee H.J., Yim H.S., Lee M.G., Sohn J.W., Yoon Y.K. (2022). In vitro synergistic antimicrobial activity of a combination of meropenem, colistin, tigecycline, rifampin, and ceftolozane/tazobactam against carbapenem-resistant *Acinetobacter baumannii*. Sci. Rep..

[307] Mansour H., Ouweini A.E.L., Chahine E.B., Karaoui L.R. (2021). Imipenem/cilastatin/relebactam: A new carbapenem beta-lactamase inhibitor combination. Am. J. Health Syst. Pharm..

[308] Yahav D., Giske C.G., Gramatniece A., Abodakpi H., Tam V.H., Leibovici L. (2020). New beta-Lactam-beta-Lactamase Inhibitor Combinations. Clin. Microbiol. Rev..

[309] Zhanel G.G., Lawrence C.K., Adam H., Schweizer F., Zelenitsky S., Zhanel M., Lagace-Wiens P.R.S., Walkty A., Denisuik A., Golden A. (2018). Imipenem-Relebactam and Meropenem-Vaborbactam: Two Novel Carbapenem-beta-Lactamase Inhibitor Combinations. Drugs.

[310] Mouktaroudi M., Kotsaki A., Giamarellos-Bourboulis E.J. (2022). Meropenem-vaborbactam: A critical positioning for the management of infections by Carbapenem-resistant Enterobacteriaceae. Expert Rev. Anti-Infect. Ther..

[311] Castanheira M., Doyle T.B., Kantro V., Mendes R.E., Shortridge D. (2020). Meropenem-Vaborbactam Activity against Carbapenem-Resistant Enterobacterales Isolates Collected in U.S. Hospitals during 2016 to 2018. Antimicrob. Agents Chemother..

[312] Zhanel G.G., Lawson C.D., Adam H., Schweizer F., Zelenitsky S., Lagace-Wiens P.R., Denisuik A., Rubinstein E., Gin A.S., Hoban D.J. (2013). Ceftazidime-avibactam: A novel cephalosporin/beta-lactamase inhibitor combination. Drugs.

[313] Mawal Y., Critchley I.A., Riccobene T.A., Talley A.K. (2015). Ceftazidime-avibactam for the treatment of complicated urinary tract infections and complicated intra-abdominal infections. Expert Rev. Clin. Pharmacol..

[314] Thompson C.A. (2015). Ceftazidime with beta-lactamase inhibitor approved for complicated infections. Am. J. Health Syst. Pharm..

[315] Stein G.E., Smith C.L., Scharmen A., Kidd J.M., Cooper C., Kuti J., Mitra S., Nicolau D.P., Havlichek D.H. (2019). Pharmacokinetic and Pharmacodynamic Analysis of Ceftazidime/Avibactam in Critically Ill Patients. Surg. Infect..

[316] Torres A., Rank D., Melnick D., Rekeda L., Chen X., Riccobene T., Critchley I.A., Lakkis H.D., Taylor D., Talley A.K. (2019). Randomized Trial of Ceftazidime-Avibactam vs Meropenem for Treatment of Hospital-Acquired and Ventilator-Associated Bacterial Pneumonia (REPROVE): Analyses per US FDA-Specified End Points. Open Forum Infect. Dis..

[317] Pouya N., Smith J.E., Hudson C.S., Teran N.S., Tam V.H. (2024). In vitro evaluation of using ceftazidime/avibactam against carbapenem-resistant *Acinetobacter baumannii*. J. Glob. Antimicrob. Resist..

[318] Ransom E., Bhatnagar A., Patel J.B., Machado M.J., Boyd S., Reese N., Lutgring J.D., Lonsway D., Anderson K., Brown A.C. (2020). Validation of Aztreonam-Avibactam Susceptibility Testing Using Digitally Dispensed Custom Panels. J. Clin. Microbiol..

[319] Al Musawa M., Bleick C.R., Herbin S.R., Caniff K.E., Van Helden S.R., Rybak M.J. (2024). Aztreonam-avibactam: The dynamic duo against multidrug-resistant gram-negative pathogens. Pharmacotherapy.

[320] Sader H.S., Kimbrough J.H., Doyle T.B., Winkler M.L., Castanheira M. (2025). Frequency, Antimicrobial Susceptibility, and Molecular Characterization of Carbapenem-Resistant Enterobacterales Stratified by United States Census Divisions: Results From the INFORM Program (2018–2022). Open Forum Infect. Dis..

[321] Wu Y., Chen J., Zhang G., Li J., Wang T., Kang W., Zhang J., Sun H., Liu Y., Xu Y. (2024). In-vitro activities of essential antimicrobial agents including aztreonam/avibactam, eravacycline, colistin and other comparators against carbapenem-resistant bacteria with different carbapenemase genes: A multi-centre study in China, 2021. Int. J. Antimicrob. Agents.

[322] Dumbleton J.T., Shah A.P., Ho B.M., Singh N., de Souza H., Smith N.M. (2025). Pharmacodynamics of aztreonam/ceftazidime/avibactam and polymyxin B versus New Delhi MBL-producing *Acinetobacter baumannii*. JAC-Antimicrob. Resist..

[323] Coppola N., Maraolo A.E., Onorato L., Scotto R., Calò F., Atripaldi L., Borrelli A., Corcione A., De Cristofaro M.G., Durante-Mangoni E. (2022). Epidemiology, mechanisms of resistance and treatment algorithm for infections due to carbapenem-resistant gram-negative bacteria: An expert panel opinion. Antibiotics.

[324] Tamma P.D., Aitken S.L., Bonomo R.A., Mathers A.J., Van Duin D., Clancy C.J. (2022). Infectious Diseases Society of America guidance on the treatment of AmpC β-lactamase–producing Enterobacterales, carbapenem-resistant *Acinetobacter baumannii*, and Stenotrophomonas maltophilia infections. Clin. Infect. Dis..

[325] Tsuji B.T., Pogue J.M., Zavascki A.P., Paul M., Daikos G.L., Forrest A., Giacobbe D.R., Viscoli C., Giamarellou H., Karaiskos I. (2019). International consensus guidelines for the optimal use of the polymyxins: Endorsed by the American college of clinical pharmacy (ACCP), European society of clinical microbiology and infectious diseases (ESCMID), infectious diseases society of America (IDSA), international society for anti-infective pharmacology (ISAP), society of critical care medicine (SCCM), and society of infectious diseases pharmacists (SIDP). Pharmacother. J. Hum. Pharmacol. Drug Ther..

[326] Rafailidis P., Panagopoulos P., Koutserimpas C., Samonis G. (2024). Current therapeutic approaches for multidrug-resistant and extensively drug-resistant *Acinetobacter baumannii* infections. Antibiotics.

[327] Tamma P.D., Aitken S.L., Bonomo R.A., Mathers A.J., van Duin D., Clancy C.J. (2022). Infectious Diseases Society of America 2022 Guidance on the Treatment of Extended-Spectrum beta-lactamase Producing Enterobacterales (ESBL-E), Carbapenem-Resistant Enterobacterales (CRE), and *Pseudomonas aeruginosa* with Difficult-to-Treat Resistance (DTR-P. aeruginosa). Clin. Infect. Dis..

